# Onset and nature of flow-induced vibrations in cerebral aneurysms via fluid–structure interaction simulations

**DOI:** 10.1007/s10237-022-01679-x

**Published:** 2023-03-02

**Authors:** David A. Bruneau, Kristian Valen-Sendstad, David A. Steinman

**Affiliations:** 1 Department of Mechanical and Industrial Engineering, University of Toronto, Toronto, ON Canada; 2 Department of Computational Physiology, Simula Research Laboratory, Oslo, Norway

**Keywords:** Aneurysm, Biological vibration, High-frequency wall motion, Fluid–structure interaction, Flow instability, Spectral analysis, Hemodynamics

## Abstract

Clinical, experimental, and recent computational studies have demonstrated the presence of wall vibrations in cerebral aneurysms, thought to be induced by blood flow instability. These vibrations could induce irregular, high-rate deformation of the aneurysm wall, and potentially disrupt regular cell behavior and promote deleterious wall remodeling. In order to elucidate, for the first time, the onset and nature of such flow-induced vibrations, in this study we imposed a linearly increasing flow rate on high-fidelity fluid–structure interaction models of three anatomically realistic aneurysm geometries. Prominent narrow-band vibrations in the range of 100–500 Hz were found in two out of the three aneurysm geometries tested, while the case that did not exhibit flow instability did not vibrate. Aneurysm vibrations consisted mostly of fundamental modes of the entire aneurysm sac, with the vibrations exhibiting more frequency content at higher frequencies than the flow instabilities driving those vibrations. The largest vibrations occurred in the case which exhibited strongly banded fluid frequency content, and the vibration amplitude was highest when the strongest fluid frequency band was an integer multiple of one of the natural frequencies of the aneurysm sac. Lower levels of vibration occurred in the case which exhibited turbulent-like flow with no distinct frequency bands. The current study provides a plausible mechanistic explanation for the high-frequency sounds observed in cerebral aneurysms, and suggests that narrow-band (vortex-shedding type) flow might stimulate the wall more, or at least at lower flow rates, than broad-band, turbulent-like flow.

**Supplementary Information:**

The online version contains supplementary material available at 10.1007/s10237-022-01679-x.

## Introduction

Cerebral aneurysms are present in over 3% of the human population (Li et al. 2013; Vlak et al. 2011), and those with cerebral aneurysms have a ~ 30% lifetime risk of rupture (Korja et al. 2014). While some general risk factors are established, such as aneurysm size and family history (Korja et al. 2014), there is a need for more specific rupture risk predictors based on the actual mechanics of rupture (Turjman et al. 2014). There has been much interest in using Computational Fluid Dynamics (CFD) to model cerebral aneurysm hemodynamics, and consequently, various fluid mechanical metrics have been proposed to predict cerebral aneurysm rupture (Meng et al. 2014). Recently, high-fidelity CFD has shown that cerebral aneurysms can exhibit flow instability, with some, but not all cerebral aneurysms exhibiting mild, periodic flow instability to turbulent-like, highly unstable flow (Valen-Sendstad and Steinman 2014). While cerebral aneurysm hemodynamics drive the wall remodeling and do appear to correlate with rupture status (Meng et al. 2014), cerebral aneurysm growth and rupture are clearly solid mechanical phenomena.

Cerebral aneurysms are known to produce sound at frequencies in the range of 150–800 Hz (Ferguson 1970; Kurokawa et al. 1994) which Ferguson suggested was caused by flow-induced vibration of the aneurysm wall. Recently, experimental studies have reproduced this phenomenon using in vitro models (Balasso et al. 2019). Aneurysm wall vibration has not, however, been examined with modern fluid–structure interaction (FSI) simulations, except for a recent proof-of-concept study on a single aneurysm case at a single steady flow rate (Souche et al. 2022). The reasons for this may be that previous FSI software was unable to simulate high-frequency vibration, or previous thinking that blood flow in aneurysms is laminar, i.e., not experiencing high-frequency pressure fluctuations. With the recent understanding that blood flow in cerebral aneurysms can be unstable (Valen-Sendstad et al. 2011), and that flow instability can cause aneurysm wall vibration (Balasso et al. 2019; Souche et al. 2022), interest in wall vibration has been renewed as a potential cause of remodeling or rupture of aneurysms, as suggested by the first studies that identified aneurysm sounds (Ferguson 1970). Vibration has been shown to result in poststenotic dilation (Boughner and Roach 1971), and can cause disruption of mural cell function (Bittle 1994; Cho et al. 2011).

The mechanism of wall vibration and the type of flow instability driving it are not well understood. It is possible that vibration is driven by increasing turbulence (Ferguson 1970), or by resonance due to vortex-shedding at Reynolds numbers (Re) close to the onset of flow instability (Aaslid and Nornes 1984; Foreman and Hutchison 1970). Previous experimental and computational studies measured wall vibration in cerebral aneurysm models at a few selected flow rates (Balasso et al. 2019; Souche et al. 2022); however, it is known that mechanical resonance occurs at discrete frequencies. The frequency content of flow instability obviously depends on the flow rate, and vibration is thought to be stimulated by an increased presence of flow instabilities at specific frequencies. It is possible that resonant frequencies could be missed by considering only a few discrete flow rates, and that a slowly increasing flow rate would circumvent this issue. In particular, we hypothesize that the wall may vibrate most, not necessarily at the highest flow rates, but at the flow rates which induce more spectral power at frequencies close to a certain resonant frequency of the wall, or at an integer multiple of those frequencies. Furthermore, previous studies of aneurysm wall vibration have not investigated the nature of vibration modes of aneurysms, which may help to understand which aneurysm geometries are more prone to vibration. Therefore, the objective of this study was to generate varying levels of flow instability in three aneurysm geometries using FSI simulations with a slowly ramped flow rate, and for the first time computationally show how flow rate affects the frequencies, amplitudes, and modes of aneurysm wall vibration.

## Methods

### Ramped velocity inflow condition

**Fig. 1:**
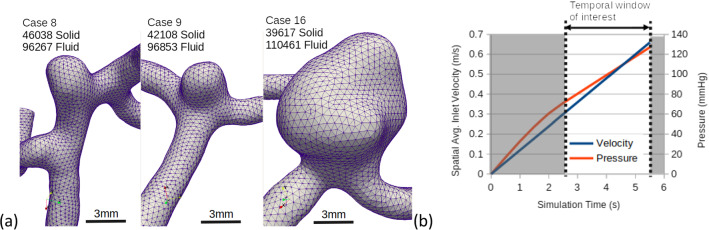
Model inputs: **a** meshed domains for the three aneurysm cases, indicating number of finite elements in solid and fluid domains; **b** spatial average inlet velocity and pressure applied to the inner wall as a function of time, for all cases. Note the “temporal window of interest,” on which the Results focus

Three cerebral aneurysm geometries representing stable flow (Case 8), mildly unstable flow (Case 9), and highly unstable flow (Case 16), as previously characterized by (Khan et al. 2015; Valen-Sendstad and Steinman 2014) at a nominal physiological flow rate, were subjected to a linearly increasing flow rate, starting at 0 m/s (i.e., a “ramp”) at the inlet. The minimum and maximum flow rates for the temporal window of interest were based on the cycle-averaged mean velocity in the Middle Cerebral Artery (MCA), and the peak systolic flow velocity in the MCA as predicted by a 30% damped older–adult flow waveform (Hoi et al. 2010). A tolerance of 20% was added as a factor-of-safety, because flow is known to vary by at least 10% within an individual (Liu et al. 2021), and peak flow in the MCA may be underestimated by a 30% damped waveform, therefore, the temporal window of interest ranged from a spatial average inlet velocity of 0.30 m/s (i.e., 0.37 m/s  − 20%) to 0.66 m/s (i.e., 0.55 m/s + 20%). This inlet velocity range corresponded with an inlet Reynolds number of 166 to 370 in Case 8, 218 to 486 in Case 9, and 228 to 508 in Case 16. It was verified that the minimum velocity in the temporal window of interest induced stable flow in all cases in a previous CFD study (Bruneau et al. 2021), to ensure that no transient effects from initialization influenced the onset of instability and subsequent wall vibration. The slope of the ramp, and therefore the length of the simulation, was chosen to result in a maximum variation of 25% in inlet velocity over the length of a cardiac cycle (0.95 s) during the temporal window of interest, to allow time for possible resonant phenomena to develop, resulting in a total physical simulation time of 5.5 s (Fig. 1b). This slope was shown to be gentle enough that the quasi-steady spectral content for the ramp resembles a “fully developed” steady flow for these same cases (Bruneau et al. 2021).

### Computational fluid–structure interaction solution strategy

The simulations were solved using a space/time centered, fully coupled, monolithic FSI solver called “turtleFSI” (Bergersen et al. 2020). A monolithic FSI solver solves the fluid and solid equations simultaneously by combining all the governing equations into a single variational form, in contrast with partitioned solvers, which iterate between two separate fluid and solid solvers each timestep. Partitioned solvers, while generally faster than monolithic solvers, can exhibit artificial “added-mass instability,” which is worsened by the small timestep size and low solid densities (Förster et al. 2007) required to simulate high-frequency wall vibration. While these instabilities have been resolved with modern coupling algorithms for some use cases (Degroote 2009), the monolithic approach prevents this instability completely at the expense of computational cost. turtleFSI solves the variational statement using a shifted Crank–Nicholson scheme (i.e., θ = 0.501) and uses Newton’s method to solve the nonlinear parts of the governing equations (Gjertsen 2017). The absolute tolerance and residual tolerance of the Newton solver were both set to 10^−6^, and the Jacobian matrix in the Newton solver was recomputed every second timestep, which was found to improve compute time while preserving the vibration response (Slyngstad 2017). A timestep of 0.340 ms (~ 3000 timesteps per second) was used, which has been shown to capture the key features of the flow in previous CFD and FSI studies (Khan et al. 2015; Souche and Valen-Sendstad 2019). The semi-implicit nature of the FSI solver allows for coarser temporal resolutions without introducing artificial dampening of flow instabilities (Gjertsen 2017; Slyngstad 2017) and allowing turbulent-like flow to develop. By turbulent-like flow, we refer to flows that are not purely laminar, with seemingly random and high-frequency velocity and pressure fluctuations, exhibiting broad-band power spectra resembling that of a turbulent flow. No turbulence model was included. The fluid velocity (power spectral density) in this aneurysm FSI implementation has been verified against CFD simulations (Souche et al. 2022). The simulations were each run on 40 Intel “Skylake” cores of a Lenovo SD530 node of the Niagara supercomputing cluster. Cases 8, 9, and 16 took, respectively, 154, 352, and 567 h to run.

### Fluid and solid model properties

The FSI meshes were generated using the Vascular Modelling Toolkit, with roughly 100,000 tetrahedral fluid elements and roughly 40,000 solid elements in each case (Fig. 1a), which translates to an average element node spacing of 0.23 mm in Case 8, 0.26 mm in Case 9 and 0.33 mm in Case 16. Quadratic elements were used, so the effective node spacing was half the average node spacing. The fluid domain had two boundary layers of total thickness 0.25 mm. For these cases, meshes with the same effective node spacing were previously shown to capture the same flow features as higher-resolution meshes with negligible differences (Khan et al. 2015). The solid was constructed as two additional layers of elements, with a total uniform wall thickness of 0.25 mm, corresponding to the average value of aneurysm wall thickness measured in cadaveric specimens (Robertson et al. 2015). The aneurysm wall was modeled using a St. Venant–Kirchhoff material model, with an elastic modulus of 1 × 10^6^ Pa and a Poisson’s ratio of 0.45, the same as in previous FSI studies of cerebral aneurysms (Bazilevs et al. 2010; Mo et al. 2020; Souche and Valen-Sendstad 2019; Torii et al. 2006; Torii et al. 2009). A Blood dynamic viscosity and density were 0.0035 Pa-s and 1000 kg/m^3^, respectively. A parabolic velocity profile was applied to the fluid at the inlet, and a zero-pressure boundary condition was applied to the fluid at the outlets. It has been shown that in quasi-steady flows, the onset of flow instability can be delayed artificially in CFD simulations, and that adding a small amount of “noise” can shift the onset of flow instability earlier, to better emulate experiments (Bergersen et al. 2019; Haley et al. 2021). Accordingly, we added a small random component of 0.001 m/s to the non-axial inlet velocities using the same methodology as in Haley et al. (2021).

### Aneurysm model boundary conditions

The deformable region of the aneurysm geometry was limited to a spherical region surrounding the aneurysm sac, with the center of the region at the center of the sac, determined using the maximum inscribed sphere. The sphere radius was determined so that it fully enclosed the aneurysm sac, with a minimum clearance of 0.5 mm. The inlet and outlet artery were made rigid and fixed in space with Dirichlet boundary conditions. Aneurysm geometry had been obtained from medical images in a pressurized state. To achieve a plausible *in vivo* geometry at physiological levels of internal pressure in the FSI simulations, the mesh was pre-deformed strategically, so that an approximate in vivo geometry and stress state were achieved during the temporal window of interest, after initializing the simulations with no pressure or internal stress at t = 0 s. The zero-pressure geometry was approximated in a separate simulation, where the desired cycle-averaged pressure of 82 mmHg (92 mmHg minus intracranial pressure (Netlyukh et al. 2015)) was applied to the medical image-based geometry, with a fluid velocity of 0.37 m/s. The reverse of the deformation from that simulation was applied to the original mesh, which was used as the approximate “zero-pressure” geometry for the ramp simulations. The internal pressure was applied as a force on the solid at the fluid–solid interface, and was varied linearly during the temporal window of interest, so that a pressure of 92 mmHg (the cycle average value (Tezduyar et al. 2008)) occurred at the same time as 0.37 m/s inlet velocity (cycle-averaged mean velocity in the MCA) and the peak systolic pressure of 120 mmHg corresponded with 0.55 m/s inlet velocity (maximum value in the MCA) (Fig. 1b). Before the temporal window of interest, increasing the pressure linearly from zero would introduce a slope discontinuity, so the pressure was increased using a sinusoid before the temporal window of interest to achieve a smooth pressure function (Fig. 1b). Intracranial pressure was assumed to be 10 mmHg, within the normal range of 5 – 15 mmHg for a healthy adult (Rangel-Castillo et al. 2008); this pressure was subtracted from the internal pressure to achieve a more realistic cerebral perfusion pressure.

### Model outputs and post-processing

For comparison with the sonic and Doppler ultrasound recordings in previous studies, spectrograms of the fluid velocity, wall displacement, and wall pressure were generated to demonstrate the onset and nature of flow instability and vibration in the three aneurysm cases, following methods presented previously (Natarajan et al. 2020). Specifically, 15 windows were used for the short-time Fourier transform (i.e., a window length of 992 timesteps), and a window overlap of 75% was used, resulting in a 1.48 Hz frequency resolution of and 0.168 s time resolution. This choice of windowing resulted in good frequency resolution that clearly showed the frequency bands (local minima and maxima) in the fluid velocity and wall displacement, while maintaining adequate temporal resolution. Power spectrum scaling (spectrogram units = (input units)^2^) was used to generate the spectrograms, which were then log-scaled. Spectrograms were calculated at each node in the spherical deformable region of the simulation using the magnitude of the fluid velocity for the fluid nodes contained within this spherical region, wall displacement, and fluid pressure at the wall. The velocity, displacement, and pressure were high-pass filtered above 25 Hz, because frequencies below 25 Hz are present in the driving flow waveform (Khan et al. 2017; Natarajan et al. 2020). Nodal spectrograms for all nodes in the deformable region were averaged to create the representative fluid velocity, wall displacement, and wall pressure spectrograms.

Vibration mode shapes were obtained by filtering the displacement components of the outer surface nodes with a Butterworth band-pass filter. The band-pass frequencies were determined by positioning the division between bands at local minimum values from the spectrogram. Overall fluid velocity fluctuation and vibration amplitudes were calculated using windowed Root-Mean Squared (RMS) amplitude of the x, y, and z component of the hi-pass filtered displacement at each node (> 25 Hz, i.e., frequencies above those in the driving flow waveform (Khan et al. 2017)), then taking the magnitude. A flat window was used and with a window length of 250 timesteps, to provide fine temporal resolution, then the 99^th^ percentile spatial value was plotted, because this is considered to be more robust than using a maximum (Speelman et al. 2008). The same process was repeated to determine amplitudes for prominent frequency bands, using band-pass filtered displacement. To assess the instability of flow structures, Q-criterion was calculated at 3 successive increments, 10 timesteps apart.

## Results

### Qualitative description of fluid flow and vibration

**Fig. 2:**
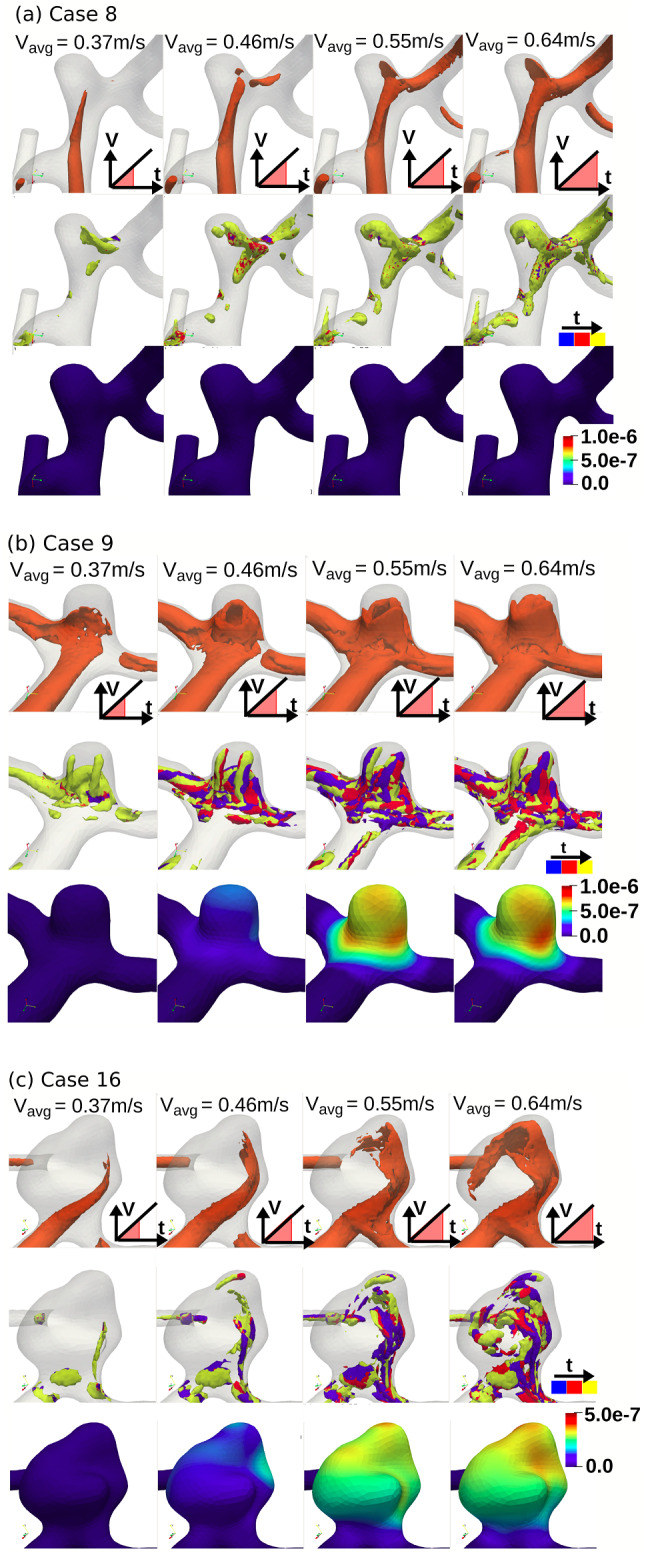
Fluid flow and wall vibration phenomena for Case 8 (**a**), Case 9 (**b**), and Case 16 (**c**) at progressive inlet velocities of 0.37 m/s, 0.46 m/s 0.55 m/s, and 0.64 m/s. For each case, the top row shows the progression of the 0.5 m/s fluid velocity isosurface. Each panel in the middle row shows vortex cores (Q = 100,000) from three steps taken over a range of 20 timesteps (or ~ 14 ms) colored blue, red, then yellow. The bottom row shows wall vibration amplitude > 25 Hz of the outer wall surface (in m)

Qualitatively, the three cases exhibited different flow phenomena (Fig. 2). Case 8 exhibited lower fluid velocity in the aneurysm sac, while Cases 9 and 16 exhibited higher fluid velocity in the aneurysm sac, as evidenced by the isosurface extending deeper into the sac during systole (Fig. 2b, c). Accordingly, Case 8 exhibited no flow instability over the entire simulation, indicated by the large, smooth structure of the vortex cores that remain consistent in shape at all inlet velocities (Fig. 2a, second row). While Cases 9 and 16 had no flow instability at an inlet velocity of 0.37 m/s (Fig. 2b, c, second row, panel 1), these cases exhibited flow instability at inlet velocities of 0.46 m/s and higher, with smaller vortex cores showing considerable motion between closely spaced timesteps (Fig. 2b,c, second row, panels 2–4). Consequently, Cases 9 and 16 started vibrating at the onset of flow instability (Fig. 2b,c, third row), while Case 8 did not vibrate (Fig. 2a, third row). Interestingly, the distribution of wall vibration varied throughout the simulations, with the location of maximum vibration amplitude at visibly different locations on the sac in the final four panels (Fig. 2b,c, third row). The maximum amplitude of vibration was similar in Case 9 (0.86 μm) and Case 16 (0.66 μm) but the amplitude in Case 9 was considerably higher at lower flow rates.

### Spectral analysis

**Fig. 3:**
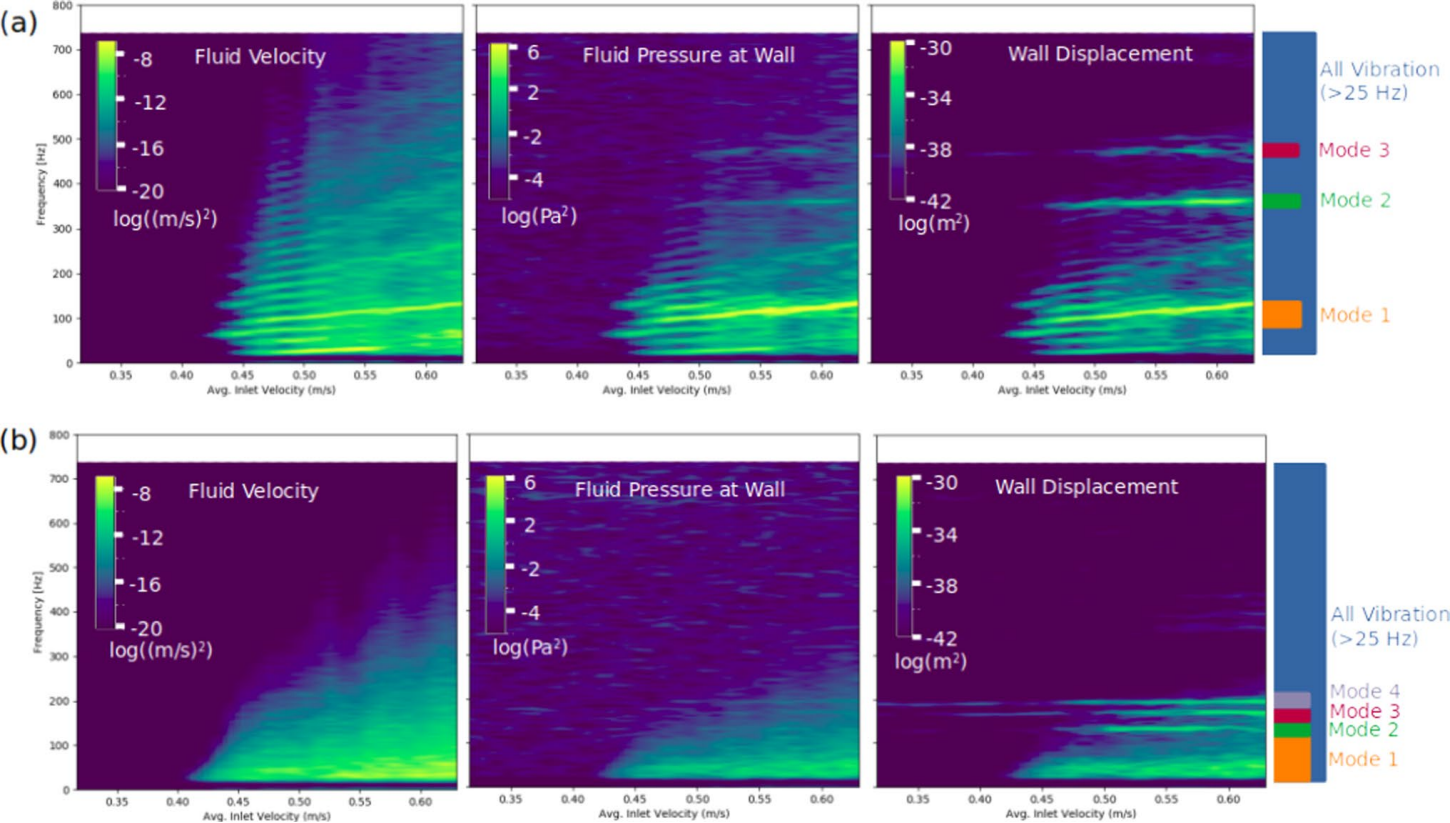
Spectrograms of fluid velocity (left), wall displacement (middle), and fluid pressure at the wall (right) for Case 9 (**a**) and Case 16 (**b**). In the spectrograms, yellow represents a high level of power while dark blue represents a lower level of power

Due to the lack of flow instability and wall vibration in Case 8, the remaining figures only show Cases 9 and 16. In both cases, the progression of flow instability can be seen in the fluid velocity spectrograms (Fig. 3): at low inlet velocity there was no flow instability present (all dark blue), then the transition between stable and unstable flow is shown by the start of the green-yellow region at around v = 0.4 m/s in both cases. Also, the intensity of flow instability generally increased as inlet velocity increased in both cases. The fluid spectrogram for Case 9 exhibited spectral banding from the onset of instability until v = 0.49 m/s, indicating harmonic, mild flow instability, which later transitioned into broader band instability but retaining a strong narrow band with an increasing frequency of 95–135 Hz. The fluid spectrogram for Case 16 shows an immediate transition to broad-band flow instability, with the instability strengthening as inlet velocity increased.

The wall displacement spectrograms share some characteristics with the fluid velocity spectrograms, but with additional high-frequency bands (not present in the fluid) superimposed. These specific narrow bands represent the vibration of the whole aneurysm sac at resonant modes with consistent frequencies, visible clearly at 360 Hz (Mode 2) and 475 Hz (Mode 3) in Case 9 and at lower frequencies of 135 Hz (Mode 2), 165 Hz (Mode 3) and 190 Hz (Mode 4) in Case 16. In both cases, wall fluid pressure spectrograms shared characteristics from both the fluid velocity and wall displacement spectrograms, with the wall modes faintly visible. In Case 9, Mode 1 (95–135 Hz) of the wall displacement mirrored the strongest band in the fluid.

### Mode shapes

**Fig. 4:**
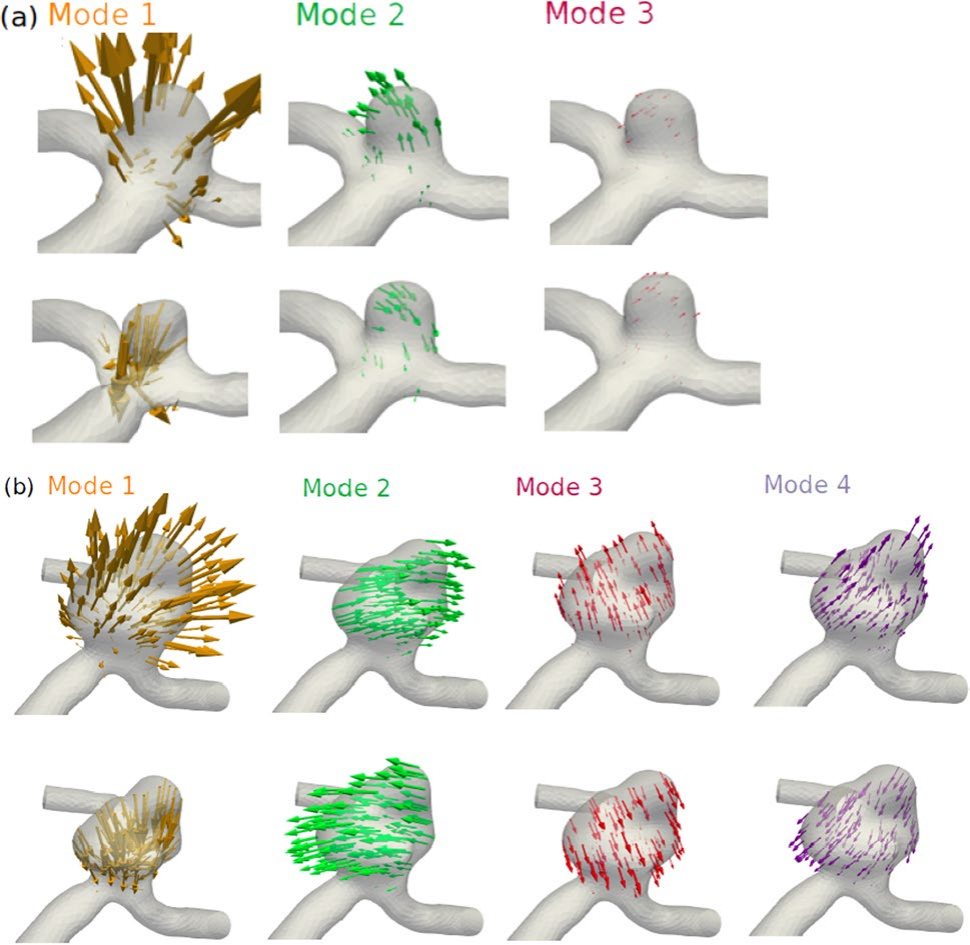
Mode shapes for Case 9 (**a**) and Case 16 (**b**), with motion vectors amplified by a factor of 1000. The top and bottom panels for each case represent the two extremes of the motion for each mode

Cases 9 and 16 had 3–4 distinct mode shapes of the sac in different directions, which remained consistent in direction and frequency as fluid velocity increased (Fig. 4). In Case 9, the band-pass filtering frequency ranges were 95–140 Hz for Mode 1, 347-375 Hz for Mode 2 and 465–490 Hz for Mode 3, while in Case 16, the frequency ranges were 25–120 Hz for Mode 1, 120–150 Hz for Mode 2, 150–180 Hz for Mode 3 and 180–205 Hz for Mode 4. Qualitatively, Mode 1 in both cases could be described as a “folding” or expansion/contraction motion of the sac, while the higher frequency modes were motions of the entire sac, where the sac “rocked” back and forth. Notably, the higher frequency Modes 2 and above varied less in frequency than Mode 1 in both cases. In Case 16, all modes occurred at lower frequencies (~ 40 Hz, 135 Hz, 175 Hz, and 190 Hz) than in Case 9 (~ 110 Hz, 360 Hz, 470 Hz). In Case 16, due to the wider band used for filtering, Mode 1 was mixed with lesser, localized “fluttering” vibrations that mimicked the frequency content of the fluid. The locations of the “fluttering” vibration varied over time, with larger amplitudes occurring at different locations on the sac at different times.

### Vibration amplitudes

**Fig. 5:**
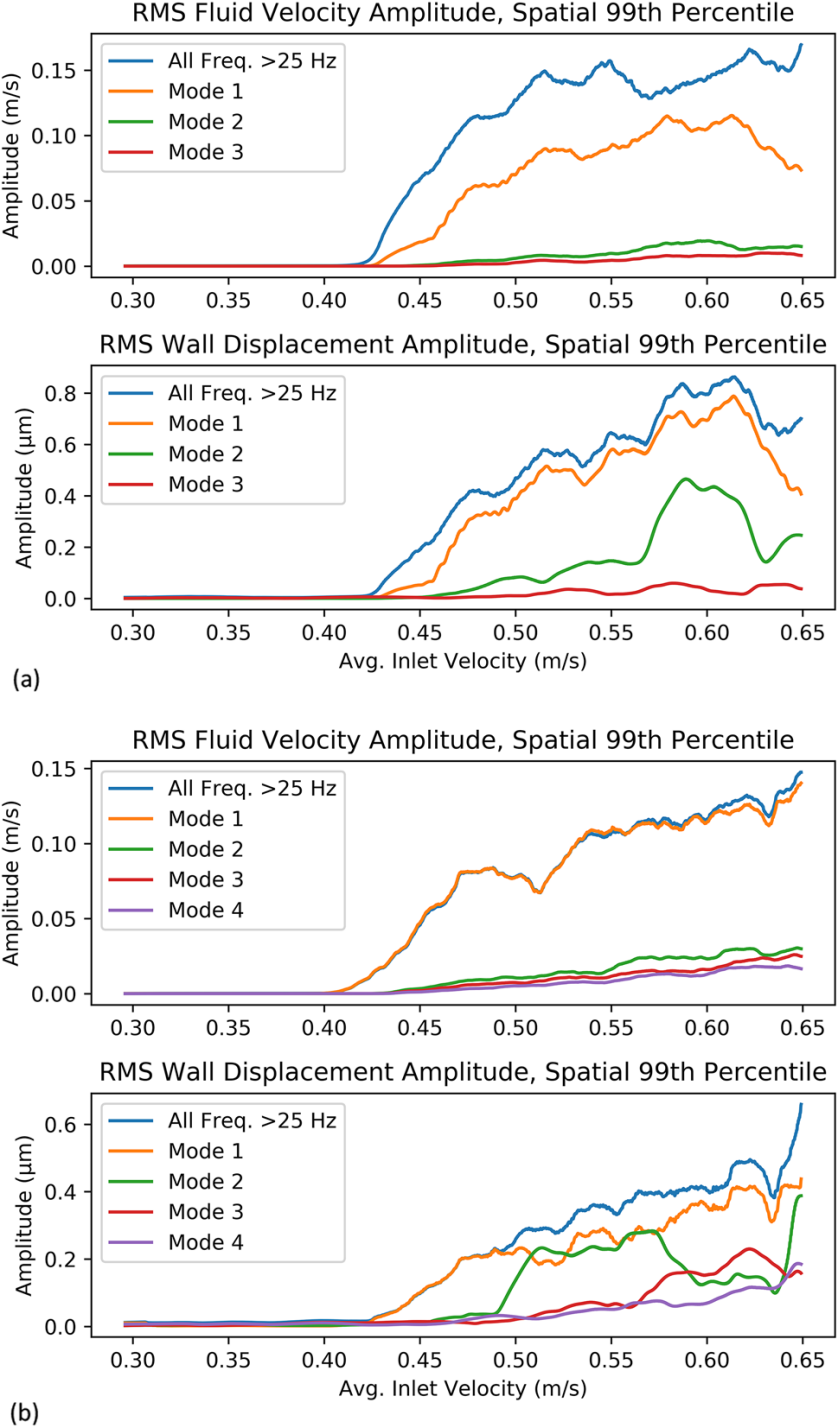
Fluid velocity and wall displacement amplitude at various frequency bands for Case 9 (**a**) and Case 16 (**b**). The RMS amplitude was calculated for all points; the 99th percentile value in space was taken

To illustrate how the amplitude of flow instability within a specific frequency band affected the amplitude of vibration of different modes, the amplitude of fluid velocity and wall displacement in specific frequency bands was plotted (Fig. 5), taking the 99^th^ percentile spatial value. In both cases, the fluid velocity and overall wall displacement amplitude (> 25 Hz) generally increased with time. In Case 16, the maximum amplitude of wall displacement for Mode 2 was 88% of the maximum amplitude of the Mode 1, while in Case 9, the maximum amplitude of Mode 2 was 59% the maximum amplitude of Mode 1. In contrast, the fluid velocity amplitudes at the frequencies of Modes 2 and above were much lower than in Mode 1 (the maximum amplitude of Mode 2 fluid velocity was 22% of the maximum amplitude of Mode 1 in Case 16, and 17% in Case 9), indicating that the wall displacement has more high-frequency content than the fluid velocity. In both cases, the Mode 2 (360 Hz in Case 9, 135 Hz in Case 16) reached a higher amplitude than Mode 3 and 4, reaching nearly 0.5 um in Case 9 and 0.4 um in Case 16. These amplitudes can be interpreted as a proxy for energy; in both cases, the aneurysm wall exhibited higher energy at higher frequencies than the flow instability, which was more energetic at lower frequencies.

## Discussion

### Mild and turbulent-like flow instability cause wall vibrations

The purpose of the current study was to better understand the nature of vibration in cerebral aneurysms by using a simplified numerical experiment. A variety of flow phenotypes, ranging from stable, to mildly unstable, to highly unstable flow, were generated using three aneurysm geometries by slowly increasing the flow rate within physiological levels. The flow phenomena observed were consistent with previous CFD studies of the same cases (Khan et al. 2015; Valen-Sendstad and Steinman 2014). Rostam-Alilou et al. also recently performed FSI of idealized intracranial aneurysm and atherosclerosis models under steady flow conditions; however, they presented oscillatory phenomena that did not appear consistent with the physics of vortex-shedding or turbulent-like flows at physiological Reynolds numbers, possibly owing to their use of a partitioned FSI solver. This further emphasizes the importance of taking care with numerical solvers and settings for these challenging coupled problems. Our study confirms the presence of wall vibrations, as shown in Souche et al. (2022), and is the first to show that wall vibrations can occur during both mildly unstable flow (with highly banded spectral content) and highly unstable flow with broad-band character. Vibrations did not occur during stable flow. It was also found that the amplitude of vibration of cerebral aneurysms generally increased as flow rate increased. However, the relationship between flow rate and vibration amplitude was not linear, and to further explain the link between flow instability and wall vibration, we must first describe the type of vibrations we observed.

### Fundamental modes of vibration of the aneurysm sac could explain previously observed aneurysm sounds

**Fig. 6:**
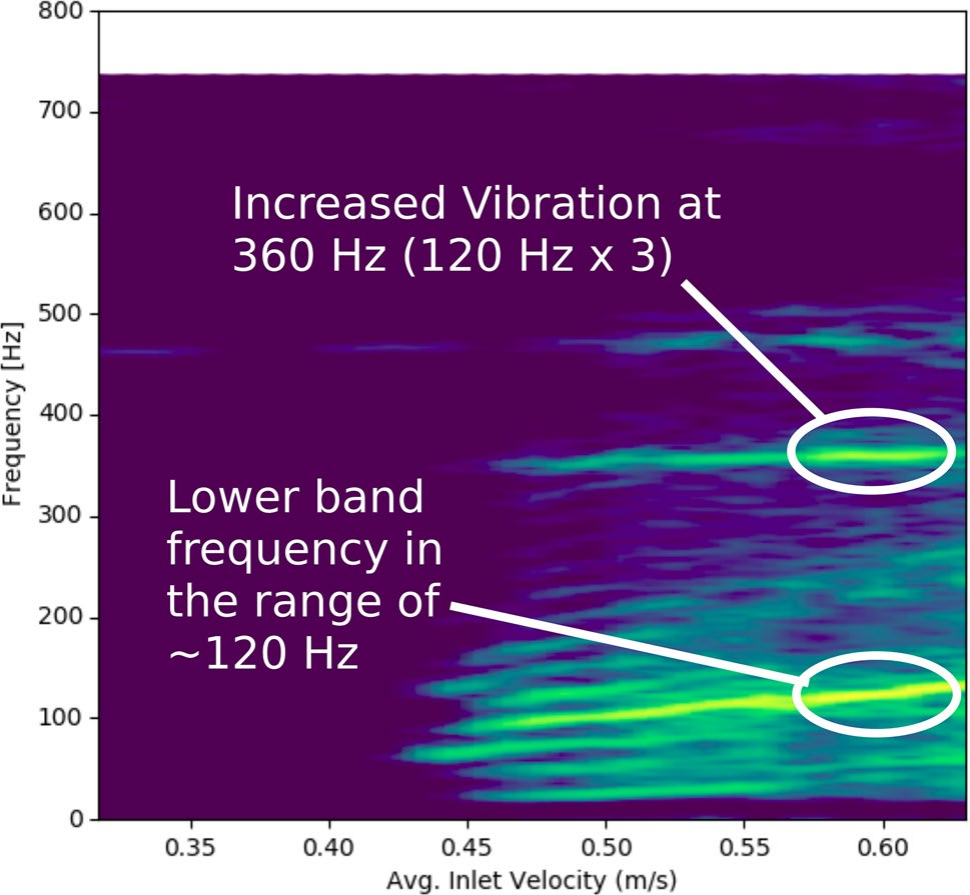
Spectrogram of Case 9 wall displacement, showing increased vibration of the 360 Hz mode when the lower-frequency band reached 120 Hz

In the current study, wall vibrations were shown to consist mostly of motions (modes) of the entire aneurysm sac, with some lesser, localized fluttering motions that mirrored the frequency content of the fluid. The frequencies of the higher modes (Modes 2 and above) appeared to be independent of the fluid frequency content and occurred at frequencies ranging from 130 to 480 Hz, in the same range as aneurysm sounds recorded in the literature (Aaslid and Nornes 1984; Kosugi et al. 1983; Kurokawa et al. 1994; Sekhar and Wasserman 1984). Further, Sekhar and Wasserman (1984) decomposed the aneurysm sounds into “bruits,” describing noise spread over a range of frequencies and “spikes” occurring at a single, distinct frequency. The spikes were described as a musical ringing, within the range of 150–800 Hz (Sekhar and Wasserman 1984). Our results may provide further insight into the mechanism behind these sounds. Based on our results, we hypothesize that “bruits” are caused by the random, localized vibration that mirrors the frequency content of the fluid, while the “spikes” are the modes of the whole sac at natural frequencies, which were represented by a single narrow-band frequency in the spectrum of the two cases with vibration in the current study. Our findings provide a reasonable explanation for the mechanism of aneurysm vibration, about which Sekhar et al. (1990) noted there was “…disagreement in the literature as to whether the phenomenon of aneurysmal vibration is due to turbulent flow, resonance, vortex-shedding, or a hydrodynamic whistle mechanism.” In our study, harmonic vortex-shedding, seen at the onset of flow instability in Case 9 (Fig. 3), caused some vibration, while in both cases, broad-band flow instability at higher flow rates caused high-frequency wall vibrations at a variety of resonant modes. In the current study, the amplitude of the higher aneurysm sac modes did not increase linearly with increased flow rates, nor were they predicted solely by the spectral content of the fluid at the same frequency of the mode. Rather, it appears that fluid spectral content at lower frequencies drove higher frequency modes of the aneurysm at higher frequency multiples (superharmonic resonance). For example, in Case 9, when the prominent band in the fluid reached a frequency of 120 Hz, Mode 2 at 3 × that frequency (360 Hz) exhibited greatly increased vibration, which subsided when Mode 1 increased beyond 120 Hz (Fig. 6). Of course, there are differences between simulated wall vibrations and clinical recordings of aneurysms. In clinical studies, acoustic signals from aneurysms were recorded via stethoscope or microphone (Kosugi et al. 1983; Kurokawa et al. 1994; Sekhar et al. 1990; Sekhar and Wasserman 1984). The sounds would need to propagate through tissue, which would suppress certain frequencies and reduce the overall amplitude (Salman and Yazicioglu 2017). We speculate that the wall pressure spectrograms in the current study are most representative of acoustic recordings, but with narrower spectral bands as there were no acoustic transmission effects. Other studies recorded signals from aneurysms using Doppler ultrasound (Aaslid and Nornes 1984), which was intended to capture the fluid velocity by filtering out the dominant, low-frequency wall motion. However, these signals were likely composed of both the fluid velocity and high-frequency wall motion.

### Mechanistic explanation of results

In our study, aneurysm vibration Mode 1 was distinctly different than Modes 2 and above, and we can offer a possible explanation for the nature of these modes with mechanical vibration theory. Modes 2 and above occurred at higher frequencies (130–470 Hz) and were isovolumetric, while Mode 1 occurred at lower frequencies (25–130 Hz), displaced fluid from the sac, and mirrored the frequency content of the fluid. As such, Mode 1 was likely highly damped by displacement of internal fluid, while Modes 2 and above were not. Mode 1 resembled the radial inflation mode of the idealized spherical aneurysm examined in Shah and Humphrey 1999, while Modes 2 and above, consisting of “rocking” motions of the entire sac, have not been shown in any previous study of aneurysm vibration. In the simplest 1D model of vibration, natural frequencies increase with increased stiffness, and decrease with increased mass. Our results suggest vibration stiffness of an aneurysm arises from both bending stiffness (like a beam) and internal pressurization (like a membrane). The frequencies of Modes 2 and above, while fairly consistent in time, rose by ~ 10 Hz from the onset of flow instability to the end of the simulation, thought to be due to the increasing pressure as the inlet flow rate increased, which would increase the stiffness and result in slightly higher resonant frequencies. However, this higher stiffness may be offset by the sac mass increasing with higher pressure due to the enlarging sac being filled with more fluid. Differences in resonant frequency between cases also agree with mechanical vibration theory, as all modes in Case 9 occurred at much higher frequencies than those in Case 16. This is because the larger sac in Case 16 had more mass, while the overall bending stiffness was similar, as they were both bifurcation aneurysms with similar inlet and outlet diameters and the same wall thickness and mechanical properties. In both cases, the lowest frequency rocking mode (360 Hz in Case 9, 135 Hz in Case 16) generally exhibits the highest amplitude over time. This is also consistent with vibration theory, as the lowest frequency mode typically requires less energy to excite and the energy from flow instability is higher at lower frequencies. The fluid mechanics were also consistent with theory; in Case 9, the fluid frequency bands increased linearly with flow rate as it would in a vortex-shedding flow with a constant Strouhal number (Stansby 1976), while in Case 16 the fluid spectrum was continuous and smooth, consistent with turbulent-like flow.

### Biological implications of aneurysm wall vibration

The implications of wall vibration for aneurysm growth and rupture are not known. Aneurysm vibration could cause additional stress in the wall that weakens it over time due to extra loading (Aaslid and Nornes 1984; Ferguson 1970), or it could be that the 100–1000 Hz frequencies induced by vibration lead to very high strain rates not normally experienced by the vessel wall, causing abnormal cell function (Bittle 1994; Cho et al. 2011), even if the vibration amplitudes are minor compared to the < 25 Hz stresses induced by intramural pressure variation over the cardiac cycle. It is known that smooth muscle cells are sensitive to changes in applied loads (Humphrey and Schwartz 2021), and that the direction of wall stresses is critically important. For example, wall shear stresses can be 5 orders of magnitude lower than circumferential stresses, yet abnormal wall shear stresses are known to affect the behavior of endothelial cells and cause wall remodeling (Humphrey and Schwartz 2021). In the current study, the vibratory component of the strain was 3–4 orders of magnitude lower than the strain caused by intramural pressure variation. Additionally, vibration introduces strains in a different direction than circumferential stress, which could be further exacerbated if there is a site of aneurysm contact with other tissue or subarachnoid trabeculae.

While it is possible that vibration does not contribute meaningfully to rupture or wall degradation, a recent CFD study found that spectral banding due to flow instability correlated with the rupture status of aneurysms and theorized that this spectral banding caused increased vibration (MacDonald et al. 2022). While we only modeled two cases with vibration in the current study, Case 9 had more spectral banding throughout the simulation and vibrated at a higher amplitude, despite its smaller size compared to Case 16. While the vibration amplitudes observed in the current study were small (~ 1 μm), the amplitude of vibration may be increased by applying a more realistic pulsatile flow. While pulsatility in an aneurysm with stable flow is not thought to cause wall vibration (Shah and Humphrey 1999), fluid deceleration in diastole is known to destabilize the flow and exacerbate flow instability (Xu et al. 2020), in contrast to the stabilizing effect of the fluid acceleration in the current study, and hence a phenomenon which is known to promote increased vibration in vortex-shedding flows (Neshamar, van der A, and O’Donoghue 2022). In pulsatile flow experiments on silicone aneurysm models, Balasso et al. (2019) observed larger vibration amplitudes compared to our computational study with steady flow. They also found that the dominant vibration occurred at lower frequencies (< 100 Hz). We speculate that this is due partly to the large size of the aneurysms in that study (the sac was at least 5 × the volume of the largest aneurysm in our study), and the long unsupported length in the phantom models, while in vivo aneurysms would be supported more closely by surrounding tissue and bone, effectively reducing the unsupported length and increasing the overall stiffness. In aneurysms with peri-aneurysmal contact, the frequencies would likely be even higher due to the structure having extra support, reducing the unsupported length of the sac.

### Potential limitations

While the phenomena observed in this study are consistent with in vivo measurements of aneurysm sounds, experimental studies, and mechanical vibration theory, this is one of the first studies on aneurysm wall vibration and there are some limitations that should be examined to determine their effect on wall vibrations. First, aneurysm walls are known to be non-uniform (Acosta et al. 2021), which may influence localized wall vibration patterns or full-body mode shapes and frequencies. Also, while adequate to understand the general phenomena, the simple Dirichlet boundary conditions in the current study could be improved using Robin boundary conditions to simulate the surrounding cerebrospinal fluid and other tissue, which would add some damping as shown in Balasso et al. (2019). Additionally, aneurysm tissue is known to be viscoelastic, which would add some additional damping (Steiger et al. 1989) and reduce amplitudes of vibration and frequency of vibration. These properties should be the focus of future studies, but as damping would mainly influence the amplitude of vibration and have less of an effect on the frequency, we expect that the general phenomena elicited from our simplified models would not be markedly different. While the purpose of the current quasi-steady study was to understand flow-induced vibration over a physiological range of flow rates, future studies should incorporate pulsatile flow to better mimic in vivo conditions, which is theorized to increase the amplitude of flow instabilities and the resulting wall vibrations.

## Conclusions

The effect of flow rate on wall vibration was investigated in three cerebral aneurysm geometries using fluid–structure interaction simulations, by subjecting each geometry to a slowly increasing steady flow rate. Two cases demonstrated flow instability and vibrations, while the other case exhibited only laminar flow and did not vibrate.

- Prominent narrow-band vibrations in the range of 100–500 Hz were observed in the smaller of the vibrating cases, while the larger of the two cases exhibited narrow-band vibrations of 120–200 Hz. Fundamental modes of the aneurysm sac were responsible for the narrow-band vibrations. The higher modes of vibration exhibited more energy at higher frequencies than the fluid instability driving those vibrations, indicating that higher vibrations were being driven by lower-frequency flow instability.
- Vibrations were largest in the smaller aneurysm case, which had strongly banded fluid frequency content, and the vibration amplitude was highest at the flow rate when the most prominent fluid frequency band was an integer multiple of one of the natural frequencies of the aneurysm sac.
- Lower levels of narrow-band vibration occurred in the case which exhibited more turbulent-like flow with no distinct frequency bands in the fluid velocity.

While only three cases were investigated, the current results suggest that aneurysm sounds observed clinically are caused by flow instability exciting the fundamental modes of the aneurysm sac, with larger amplitudes present when the flow exhibits distinct frequency bands, suggesting that narrow-band (vortex-shedding) flow might be more deleterious than broad-band, turbulent-like flow.

## Supplementary Information

Below is the link to the electronic supplementary material.

Supplementary file1 (PDF 4216 kb)

## Data Availability

The data that support the findings of this study are available from the corresponding author, D.A.B., upon request.

## References

[CR1] Aaslid R, Nornes H (1984). Musical murmurs in human cerebral arteries after subarachnoid hemorrhage. J Neurosurg.

[CR2] Acosta JM (2021). Effect of aneurysm and patient characteristics on intracranial aneurysm wall thickness. Front Cardiovascular Med.

[CR3] Balasso A (2019). High-frequency wall vibrations in a cerebral patient-specific aneurysm model. Biomed Tech.

[CR4] Bazilevs Y (2010). Computational vascular fluid-structure interaction: methodology and application to cerebral aneurysms. Biomech Model Mechanobiol.

[CR5] Bergersen AW, Mortensen M, Valen-Sendstad K (2019). The FDA Nozzle benchmark: ‘In theory there is no difference between theory and practice, but in practice there is’. Int J Num Methods in Biomed Eng.

[CR6] Bergersen AW, Slyngstad A, Gjertsen S, Valen-sendstad K (2020). TurtleFSI : a robust and monolithic fenics-based fluid-structure interaction solver. J Open Source Softw.

[CR7] Bittle, Becky B. (1994) An investigation into the role of arterial wall vibration in the pathogenesis of atherosclerosis.”

[CR8] Bruneau DA (2021). Impact of flow rate on high frequency flow instabilities in intracranial aneurysms, with implications for wall vibration. In Summer Biomech, Bioeng, Biotransport Conf.

[CR9] Cho JG (2011). Tissue vibration induces carotid artery endothelial dysfunction: a mechanism linking snoring and carotid atherosclerosis?. Sleep.

[CR10] Degroote J, Bathe KJ, Vierendeels J (2009). Performance of a new partitioned procedure versus a monolithic procedure in fluid-structure interaction. Comput Struct.

[CR11] Ferguson GG (1970). Turbulence in human intracranial saccular aneurysms. J Neurosurg.

[CR12] Foreman JEK, Hutchison KJ (1970). Arterial wall vibration distal to stenoses in isolated arteries of dog and man. Circulation Res XXV.

[CR13] Förster C, Wall WA, Ramm E (2007). Artificial added mass instabilities in sequential staggered coupling of nonlinear structures and incompressible viscous flows. Comput Methods Appl Mech Eng.

[CR14] Gjertsen S (2017) Development of a verified and validated computational framework for fluid-structure interaction. University of Oslo.

[CR15] Haley AL, Valen-Sendstad K, Steinman DA (2021). On delayed transition to turbulence in an eccentric stenosis model for clean vs. noisy high-fidelity CFD. J Biomech.

[CR16] Hoi Y (2010). Characterization of volumetric flow rate waveforms at the carotid bifurcations of older adults. Phys Meas.

[CR17] Humphrey JD, Schwartz MA (2021). Vascular mechanobiology: homeostasis, adaptation, and disease. Annu Rev Biomed Eng.

[CR18] Khan MO, Valen-Sendstad K, Steinman DA (2015). Narrowing the expertise gap for predicting intracranial aneurysm hemodynamics: impact of solver numerics versus mesh and time-step resolution. Am J Neuroradiol.

[CR19] Khan MO (2017). On the quantification and visualization of transient periodic instabilities in pulsatile flows. J Biomech.

[CR20] Korja M, Lehto H, Juvela S (2014). Lifelong rupture risk of intracranial aneurysms depends on risk factors: a prospective finnish cohort study. Stroke.

[CR21] Kosugi Y (1983). Sonic detection of intracranial aneurysm and AVM. Stroke.

[CR22] Kurokawa Y, Abiko S, Watanabe K (1994). Noninvasive detection of intracranial vascular lesions by recording blood flow sounds. Stroke.

[CR23] Li M-H (2013). Annals of internal medicine prevalence of unruptured cerebral aneurysms in Chinese adults aged 35 to 75 years. Ann Intern Med.

[CR24] Liu X (2021). Identification of intra - individual variation in intracranial arterial flow by mri and the effect on computed hemodynamic descriptors. Magnet Resonance Mater Phys, Biol Med.

[CR25] MacDonald DE, Najafi M, Temor L, Steinman DA (2022). Spectral bandedness in high-fidelity computational fluid dynamics predicts rupture status in intracranial aneurysms. J Biomech Eng.

[CR26] Meng H, Tutino VM, Xiang J, Siddiqui A (2014). High WSS or Low WSS? Complex interactions of hemodynamics with intracranial aneurysm initiation, growth, and rupture: toward a unifying hypothesis. Am J Neuroradiol.

[CR27] Mo X, Meng Q, Yang X, Li H (2020). the impact of inflow angle on aneurysm hemodynamics : a simulation study based on patient-specific intracranial aneurysm models. Front Neurol.

[CR28] Natarajan T (2020). On the spectrographic representation of cardiovascular flow instabilities. J Biomech.

[CR29] Neshamar OE, van der Dominic AA, Tom O (2022). Flow-induced vibration of a cantilevered cylinder in oscillatory flow at high KC. J Fluids Struct.

[CR30] Rostam-Alilou AA, Jarrah HR, Zolfagharian A, Bodaghi M (2022). Fluid–structure interaction (FSI) simulation for studying the impact of atherosclerosis on hemodynamics, arterial tissue remodeling, and initiation risk of intracranial aneurysms. Biomech Model Mechanobiol.

[CR31] Salman HE, Yazicioglu Y (2017). Flow-induced vibration analysis of constricted artery models with surrounding soft tissue. J Acoustical Soc Am.

[CR32] Sekhar LN, Wasserman JF (1984). Noninvasive detection of intracranial vascular lesions using an electronic stethoscope. J Neurosurg.

[CR33] Sekhar LN, Sun M, Bonaddio D, Sclabassi RJ (1990). Acoustic recordings from experimental saccular aneurysms in dogs. Stroke.

[CR34] Shah AD, Humphrey JD (1999). Finite strain elastodynamics of intracranial saccular aneurysms. J Biomech.

[CR35] Slyngstad A (2017) Verification and validation of a monolithic fluid-structure interaction solver in FEniCS a comparison of mesh lifting operators. University of Oslo.

[CR36] Souche A, Kristian V-S (2019) Are high-frequency aneurysm wall vibrations of importance?” In *6th International Conference on Computational and Mathematical Biomedical Engineering*, ed. M. Oshima P. Nithiarasu, M. Ohta. 6th International Conference on Computational and Mathematical Biomedical Engineering, pp. 714–17.

[CR37] Souche A et al (2022) High-fidelity fluid structure interaction simulations of turbulent-like aneurysm flows reveals high-frequent narrowband wall vibrations: a stimulus of mechanobiological relevance? J Biomech 145(2022):111369 10.1016/j.jbiomech.2022.111369 36375263

[CR38] Speelman L, Bosboom EMH, Schurink GWH, Hellenthal FAMVI, Buth J, Breeuwer M, Jacobs MJ, van de Vosse FN (2008). Patient-specific AAA wall stress analysis: 99-percentile versus peak stress. Eur J Vasc Endovasc Surg.

[CR39] Stansby PK (1976). The locking-on of vortex shedding due to the cross-stream vibration of circular cylinders in uniform and shear flows. J Fluid Mech.

[CR40] Steiger HJ, Aaslid R, Keller S, Reulen HJ (1989). Strength, elasticity and viscoelastic properties of cerebral aneurysms. Heart Vessels.

[CR41] Torii R (2006). Computer modeling of cardiovascular fluid-structure interactions with the deforming-spatial-domain/stabilized space-time formulation. Comput Methods Appl Mech Eng.

[CR42] Torii R (2009). Fluid-structure interaction modeling of blood flow and cerebral aneurysm: significance of artery and aneurysm shapes. Comput Methods Appl Mech Eng.

[CR43] Turjman AS, Turjman F, Edelman ER (2014). Role of fluid dynamics and inflammation in intracranial aneurysm formation. Circulation.

[CR44] Valen-Sendstad K, Steinman DA (2014). Mind the gap: impact of computational fluid dynamics solution strategy on prediction of intracranial aneurysm hemodynamics and rupture status indicators. Am J Neuroradiol.

[CR45] Valen-Sendstad K (2011). Direct numerical simulation of transitional flow in a patient-specific intracranial aneurysm. J Biomech.

[CR46] Vlak MHM, Algra A, Brandenburg R, Rinkel GJE (2011). Prevalence of unruptured intracranial aneurysms, with emphasis on sex, age, comorbidity, country, and time period: a systematic review and meta-analysis. Lancet Neurol.

[CR47] Xu D et al. (2020) Nonlinear hydrodynamic instability and turbulence in pulsatile flow. Proceedings of the National Academy of Sciences of the United States of America 117(21). 10.1073/pnas.1913716117 PMC7260989 32393637

[CR50] Boughner DR, Roach MR (1971) Effect of low frequency vibration on the arterial wall. Circ Res 29(2):136–144. 10.1161/01.RES.29.2.136 10.1161/01.res.29.2.136 5105817

[CR51] Rangel-Castillo L, Gopinath S, Robertson CS (2008) Management of intracranial hypertension. Neurol Clin 26(2):521–541 10.1016/j.ncl.2008.02.003 PMC2452989 18514825

[CR52] Robertson AM, Duan X, Aziz KM, Hill MR, Watkins SC, Cebral JR (2015) Diversity in the strength and structure of unruptured cerebral aneurysms. Annu Biomed Eng 43(7):1502–1515. 10.1007/s10439-015-1252-4 10.1007/s10439-015-1252-4 PMC4497939 25632891

