# Supplementary material for: Onset and nature of flow-induced vibrations in cerebral aneurysms via fluid–structure interaction simulations
Source: Biomech Model Mechanobiol. 2023 Mar 2;22(3):761–71. doi: 10.1007/s10237-022-01679-x (PMC10167181; doi:10.1007/s10237-022-01679-x)
Supplement: Supplementary file 1 — Supplementary file1 (PDF 4216 kb) [file 10237_2022_1679_MOESM1_ESM.pdf]

High-Fidelity Fluid Structure Interaction Simulations of Turbulent-Like Aneurysm Flows Reveals High-Frequency Narrowband Wall Vibrations: A Stimulus of Mechanobiological Relevance?

Alban Souche[1], Kristian Valen-Sendstad[1*]

[1] *Department of Computational Physiology*
Simula Research Laboratory
Kristian Augusts gate 23
0164 Oslo, Norway
[*] *Corresponding author: kvs@simula.no, Telephone: +47-41000700*

Abstract

Recent high-fidelity/resolution computational fluid dynamics simulations of intracranial aneurysm hemodynamics have revealed turbulent-like flows. We hypothesized that the associated high-frequency pressure fluctuations could promote aneurysm wall vibrations. We performed fully coupled high-fidelity transient fluid structure interaction simulations between the blood flow and compliant aneurysm sac wall taking 5,000 time steps per second using a 3D patient-specific model previously shown to harbour turbulent-like flow. Our results show that the flow velocity contained fluctuations with a smooth and continuously decaying energy up to ∼160Hz, and fluctuating pressures with characteristic frequency peaks at approximately 30, 130 and 210Hz. There was a strong two-way coupling between the pressure and the wall deformation, for which the frequency spectrum showed similar characteristics, but with a narrow band peak at ∼120Hz with large regional differences in amplitude up to 80 micron. The physics of the flow is broadly consistent with clinical reports of turbulent-like flows, while the physics of the wall is consistent with reports of spectral peaks in aneurysm patients. As many aneurysms are known to harbour turbulent-like flows, wall vibrations could be a widespread phenomenon. Finally, since aneurysms are vascular pathologies by definition and many/most aneurysms

do not have endothelial cells but still display a focal remodelling, we hypothesise that vibrations and stresses within the wall itself might play a role in the mechanobiological processes of vessel wall pathology.

Keywords: Cerebral Aneurysm, Flow Instabilities, Fluid Structure Interaction, Aneurysm Wall Vibration

---

1. Introduction

It is estimated that roughly 3% of the population harbour cerebral aneurysms, which are the most common cause of subarachnoid hemorrhage (Wiebers et al., 2003). Management of aneurysms includes endovascular coiling and surgical clipping, but optimal individual decision-making is difficult as the risk of intervention can exceed the natural risk of rupture (Rinkel et al., 1998). Morphological indices have historically been used for risk of rupture stratification (Ujiie et al., 1999), but are essentially surrogates for hemodynamically induced wall shear stress (WSS) that contributes to vessel wall adaption, remodeling, and vascular pathogenesis (Malek et al., 1999). These stresses cannot be measured, but instead, routinely available medical images have been used as input to computational fluid dynamics (CFD) (Steinman et al., 2003) in the investigation of 'patient-specific' vascular pathology, e.g., retrospectively correlating WSS with disease outcome in search for a prospective clinical tool (Cebral et al., 2011). However, what constitutes an appropriate computational model has not been rigorously and/or consistently determined. There is a relatively large variability in predicted WSS (Berg et al., 2018; Valen-Sendstad et al., 2018), and several studies have therefore critically investigated and evaluated various aspects of a typical image-based modelling pipeline in the pursuit of making 'patient-specific' CFD actually *patient-specific*. As summarised and reviewed in (Steinman and Pereira, 2019), special attention has been on the role of medical image modality, segmentation, flow rates, and effects of non-Newtonian rheology. Evaluation of these modeling assumptions have in isolation provided fruitful insight into aneurysm hemodynamics, but have had limited effect on the predicted hemody-

namic indices that are thought to be of mechanobiological relevance. The above mentioned modeling assumptions definitely adds modeling complexity and precision, but accuracy remains an open question.

A broader and maybe even more relevant question is *'what model provides the most complete insight to the fundamental mechanisms of aneurysm pathophysiology?'*. The latter obviously depends on the scientific question being asked. Some have focused on the role of compliant vascular domains using fluid structure interaction (FSI), primarily investigating $\sim$1Hz aneurysm wall pulsation and the effects on WSS related quantities. For example, Torii et al. (Torii et al., 2007) primarily evaluated the effects of wall properties on wall shear stress-based risk factors and concluded that rigid wall CFD simulations might be over- or underestimating certain quantities, considering FSI simulation results as ground truth. Others (Isaksen et al., 2008) have focused on characterization of aneurysm wall tension during inflation/deflation of a cardiac cycle, drawing hypothetical links to its role in rupture. Nevertheless, consistent with the vast majority of CFD studies, those have been performed under the explicit or implicit assumption of laminar flow. In contrast, more recent investigations have shown that certain aneurysms may harbour unstable flow phenotypes ranging from 50Hz oscillatory flows (Baek et al., 2010) to vortex shedding at hundreds of Hz (Ford and Piomelli, 2012; Valen-Sendstad et al., 2012), or apparently 'turbulent' flows (Valen-Sendstad et al., 2011; Steinman et al., 2013; Valen-Sendstad and Steinman, 2014). By 'turbulent', or turbulent-like, we refer to flows that clearly deviate from a purely laminar regime, where there are rapid and seemingly random velocity and pressure fluctuations in time and space with a continuous energy transfer across scales. Such flows may exhibit certain features similar to that of turbulence, but do not necessarily exhibit all of the well-known characteristics (Tennekes and Lumley, 1972) or follow mathematical theories (Pope, 2000) of high Reynolds number homogeneous and isotropic turbulence itself. The aim of the current study was therefore to investigate whether turbulent-like flows and the associated high-frequency pressure fluctuations could give rise to aneurysm wall vibrations, by performing high-fidelity

FSI simulations at sufficiently high temporal resolution.

2. Methods

2.1. Pre-processing

We used an anatomically plausible intracranial sidewall aneurysm model with a diameter of approximately 10mm located at the C6/C7 segment of the internal carotid artery, see Figure 1. The Vascular Modeling Toolkit (Antiga et al., 2008) was used to generate a combined solid/fluid mesh with an assumed wall thickness of .25mm. The mesh consisted of approximately 150,000 tetrahedral elements with 110,000 and 40,000 elements in the fluid and solid mesh, respectively, with a corresponding average node spacing of .25mm, and .125mm. The compliant domain was restricted to the region in the vicinity of the aneurysm sac, whereas the rest of the vasculature was prescribed with rigid walls to reflect, to a first order, the particular intracranial confinement of the internal carotid artery within the skull. We assumed the wall to behave like a hyperelastic material using the St. Venant-Kirchhoff model with a Young's modulus of 1MPa, Poisson's ratio of 0.45, and a density of 1000 kg/m3 (Isaksen et al., 2008). We performed a uniaxial test (Figure 2a) and compared the results of our soft tissue model of the compliant wall against previously published experimental data of unruptured aneurysms (Robertson et al., 2015) in Figure 2b.

Our prestress calculation follows the work of (Bols et al., 2013) in obtaining the so-called zero pressure geometry. We applied a hydrostatic pressure of 50mmHg, assuming an average of 60mmHg of arterial blood pressure (Bols et al., 2013) minus 10mmHg intracranial pressure (Alperin et al., 2000) over a cardiac cycle. The zero pressure geometry is then used as initial mesh input to our FSI model that we gradually re-pressurize during the simulation to recover the patient-specific geometry. Applying a constant traction along the inner aneurysm wall (FSI interface) allows us to separate the average pressure of the vascular system from the dynamic pressure fluctuations driving and in-

duced by the blood flow itself. Figure 2c illustrates the difference between the patient-specific geometry (shaded) and the computed zero pressure geometry (inner mesh). As shown in Figure 2d, the initial stretch associated with pressurisation in the patient-specific configuration may locally reach approximately 1.15, placing the initial stress condition of the model in the lower elastic range of the constitutive law.

2.2. Numerical Simulations

The FSI simulation was performed using the open-source turtleFSI solver (Bergersen et al., 2020). In brief, turtleFSI is a fully coupled and monolithic solver defined in the classical arbitrary Lagrangian-Eulerian formulation (Wick, 2011). The solver relies on a generalized θ-scheme, where we specified $\theta = \frac{1}{2} + \Delta t$ to obtain a solution that is 2nd order accurate in time. We used the simple and efficient second-order Laplace equation for mesh lifting operations, which is most suitable for small deformations. turtleFSI is stable, accurate, and free from numerical artefacts typically associated with added-mass effect in low-order partitioned FSI implementations (Fernández, 2011). It serves as an excellent point of reference for benchmark studies precisely because it avoids added-mass effects (at the cost of speed). We used quadratic Taylor-Hood elements ($\mathbb{P}_2 - \mathbb{P}_1$) for the velocity-pressure field and quadratic $\mathbb{P}_2$ elements for the solid deformation, where the formal accuracy in space is $\mathbb{P} + 1$ in the $\mathbb{L}^2$ norm. The latter is equivalent to a mesh size of at least ∼1,2M linear tetrahedron elements, and the effective node spacing was therefore .125mm and .0625mm for the fluid and solid domains, respectively. We assumed blood to behave like an incompressible Newtonian fluid with dynamic viscosity and density of 3.5E-3 Pa.s 1.025E3 kg/m3, respectively. We prescribed a constant volumetric flow rate of 6.15 mL/s, which corresponds to the mean peak systolic flow rate for the ICA (Hoi et al., 2010) scaled according to the square of the inlet diameter (Valen-Sendstad et al., 2015)(Re=576), taking 5,000 steps per second. Relative to the prestress, we applied a constant zero pressure boundary condition at the outlets with zero-flux boundary conditions for the velocity. A validation of the implementation is

shown in the Supplemental Material A, comparing the results of turtleFSI with rigid walls against the results of a previously validated CFD solver.

We initialised the simulations by first solving the Stokes equations (same boundary conditions) to avoid artificial oscillations in the initial conditions. We then ramped up the flow and pressure (at the FSI interface) linearly over a time interval 0-0.05 seconds. Simulations were run for a total of 1 physical second, and statistics were based on the time interval 0.1-1s to allow for the artificial initial conditions to wash out.

2.3. Post-processing

Instantaneous velocity, pressure, and deformation time traces were probed at the nearest node within the fluid and solid domains to the probe locations shown in Figure 1. The fluctuating components of these quantities were further used to compute the power spectral density (PSD) using Matplotlib (Hunter, 2007). We used Welch averaging algorithm that efficiently removes the potential signal noise from the temporal signal, and further normalized the power spectrum density ad-hoc to its power at a frequency of 60Hz to better compare the signals from the different simulations. For the windowed averaging, the segment length was set to 500, signal padding specified to be 3000, with no overlap for 25 points, and a sampling frequency of 5000.

3. Results

3.1. Flow characteristics

We focus first on global variables shown in Figure 3. The flow field is characterised by a jet entering the aneurysm and propagating up to the top aneurysm wall, after which the flow field develops into a singular unsteady eddy, as shown in panels a and b. Panels c and d shows the standard deviation of the time averaged dynamic pressure, relative to the prestress. We observe a maximum of the pressure standard deviation, up to 180 Pa, at the location where the blood jet breaks against the aneurysm wall, and up to 100 Pa at the top of the vortex channelizing the blood downstream out of the aneurysm.

3.2. Characteristic temporal and spectral signatures

The temporal evolution of the fluid velocity, pressure, and solid deformation amplitudes are shown in Figure 4 a-c. The ramp up of the velocity and pressure is clearly visible $< .05$ seconds. Focusing now on results after .1 seconds, the artificial initial conditions have washed out, we can see that an unstable flow pattern has been established despite the steady inflow. Panels a and b show random fluctuations, with no discernible patterns. In contrast, the fluctuation of the aneurysm wall displacement (Figure 4c), exhibits a pseudo-randomness with repeating distinguishable patterns. The relative range of the wall deformation fluctuation reaches up to approximately 80 microns.

Figure 5 shows the PSD of the velocity, pressure, and deformation time traces presented on logarithmic (10-1500 Hz) and linear (10-300 Hz) scales, on the left and right panels, respectively. The PSD of the fluid velocity is plotted panel a shows a nearly monotonic decay as function of the frequency and do not reveal any dominant frequencies, as expected in a turbulent-like flow. The same data is shown in panel b where we observe no distinct frequency and there is essentially no energy above the tiny "bump" at 160Hz. In contrast, the pressure signals shown in panels c and d do however reveal different spectral characteristics, with noticeable peaks at approximately 30 Hz, 130 Hz, and 210 Hz. These peaks are observed, with almost identical frequencies, in the solid deformation signal shown in panels c and d. We also notice that the solid deformation signal has a much greater response to the $\sim$120 Hz frequency band, whereas it only corresponds to a secondary frequency in the pressure signal.

4. Discussion

We have for the first time shown that an anatomically plausible aneurysm model with a compliant sac can exhibit a turbulent-like flow that gives rise to quasi-random high-frequency narrowband aneurysm wall vibrations. Our computational findings demonstrate the interplay between a continuous spectrum of turbulent-like flows and characteristic pressure fluctuations that creates a strong

(two-ways) coupling between the fluid pressure and the wall deformation, and high-frequency deformation response of the aneurysm wall with characteristic narrowband peaks. The latter is of importance because the physics of the flow, continuous spectrum of flow instabilities, is consistent with clinical evidence of bruits recorded by microphone on exposed aneurysm sacs during open head surgery (Ferguson, 1970), while the physics of the wall, the distinct narrowband spikes in the wall deformation spectral signal, is consistent with spectral spikes in acoustic recordings on the eyes in patients with aneurysms (Kurokawa et al., 1994). We would therefore argue that the observed phenomenon is physiologically plausible. Secondly, in historical clinical studies that have looked for vibrations in "unselected" aneurysms, they were found in about 60% (Ferguson, 1970), and 30% in computational studies (Khan et al., 2021), so flow-induced wall vibration could be widespread. In the following, we will discuss potential implications, address the mechanobiological relevance, and contextualize the results.

4.1. Relation to others

While we are the first to demonstrate flow-induced wall vibrations in a cerebral aneurysm using computational modelling, the idea dates back much further. To the authors' knowledge, the first reports of aneurysm "turbulence" dates back to Richardson and Kofman (Richardson and Kofman, 1951) who in 1951 referred to the whirlpool in the Niagara falls as a *'magnificent example of circulation in an aneurysm'*. However, maybe Ferguson's "Turbulence in human intracranial saccular aneurysms" from 1970 was potentially more convincing where he concluded "vibration produces and accelerates degenerative changes in vascular tissue". The latter was the basis for Hung et al. who in 1975 studied a thin-shell theory applied to a spherical model under time-dependent internal pressure. Based on dynamic analysis, the authors concluded that "resonance and consequently rupture is then expected when the bruit frequency is the same as the natural frequency of the aneurysm" (Hung and Botwin, 1975). Strother et al. had in 1992 a more clinical and applied view, and said that "fluctuations

in flow are known to induce added mechanical stress, vibrations, and perhaps even resonance, all of which may contribute to aneurysm rupture" (Strother et al., 1992). Regarding the latter, we have admittedly not demonstrated any resonant effects, but that is also relatively tricky to infer resonance from a single simulation. It is not difficult to imagine that such may occur physiologically or in certain phases of a pulsatile simulation, which could ultimately affect rupture, such as Strother hypothesised. In 2010, Jou et al. (Jou and Mawad, 2010) expanded on the ideas and developed a theory for inferring aneurysm wall thickness, obviously associated with certain assumptions and limitations. They also provide a nice overview of authors having previously hypothesised aneurysm bruits and vibrations being induced by vortex shedding phenomena in the parent artery, Helmholtz resonance of the sac, etc. Nevertheless, we agree with Jou et al. that high-frequency aneurysm wall vibrations might occur, although we would argue there may be slightly different "sources" causing the vibrations. In addition to these clinical observations and theoretical considerations, Balasso et al. (Balasso et al., 2018) investigated high-frequency vibrations in an anatomically plausible aneurysm silicone model *in-vitro*, varying both flow rate and phantom elasticity properties. They reported that the frequencies had two distinct peaks, at 40-60Hz and 255-265Hz, irrespective of the flow rates, although with different amplitudes.

4.2. Mechanobiological relevance

An obvious question is what FSI simulations of wall vibrations may add to our understanding of aneurysm pathophysiology when several single-centre studies have shown differences in hemodynamics of ruptured versus unruptured aneurysms. The first problem however is that the results are inconsistent; there are for example conflicting results whether physiologically higher (Cebral et al., 2011) or lower (Xiang et al., 2011) WSS are associated with aneurysm rupture status in large retrospective studies. In an attempt to elucidate potential nuances, the community has introduced nearly 100 commonly computed hemodynamic indices (Liang et al., 2019), quantifying variants of WSS. Still, after

thousands of aneurysm flow simulations, hundreds of studies quantifying dozens of metrics, there is essentially no consensus in the literature (Yagi et al., 2019). This might indicate that there are other or more complex mechanobiological processes a computational model must incorporate. Aneurysms for instance are "diseased" by definition and it is unknown if the same mechanobiological mechanisms are involved in remodeling of aneurysms and normal healthy vessels, which is the implicit underlying assumption (Robertson and Watton, 2012), see e.g., Shojima et al. (Shojima et al., 2004). Secondly, the majority of metrics that are being computed are based on correlations between flow phenotype and gene expression based on healthy endothelium (Chien, 2007). However, many/most aneurysms don't even have intact endothelium (Frösen et al., 2004). Near wall transport may still be a relevant mechanism despite the endothelium being damaged, and is demonstrated to occur at multiple regions in the vascular system (Arzani and Shadden, 2018). Interestingly in this model (Figure 3), low and swirling flow, which is typically associated with WSS fixed points, correlates with high pressure standard deviations and vibrations. The latter may be unique for regions in the vascular system that harbour turbulent-like flows, but because of the focal remodelling, we hypothesise that the wall might sense vibrations, but admit that the potential effects on wall mechanobiology are unknown.

There is historical precedence for making the link between vibration and mechanopathobiology. Roach et al. (Roach, 1963a) made the point already in 1963 that there is a focal correlation between turbulence, bruits (sounds), vibrations, and post stenotic dilatations. Roach investigated this thoroughly in multiple regions of the cardiovascular system, but also proved the theory in animal models (Roach, 1963b). However, Roach never found histological proof of (adverse) arterial remodelling, and although vibrations caused poststenotic dilatation, the process was actually reversed when the artificial stenosis was removed. Later however, it was reported that if the dilated area grows beyond two diameters, it changes character and "may exhibit permanent aneurysmal changes" (PB, 1991). However, a recurring problem at the time was the diffi-

culties distinguishing between the effects on the endothelium versus the vessel wall. Interest in vibrations then seemed abandoned in the literature after 1993, potentially because Ojha et al. (Ojha and Langille, 1993) published a paper entitled "Evidence That Turbulence Is Not the Cause of Poststenotic Dilatation in Rabbit Carotid Arteries". However, that seems to be based on a misinterpretation of results when comparing locations of turbulent-like flows in hyper-realistic symmetrical experimental models to in-vivo animal observations. The 'sterile' and laminar experiments performed at a Reynolds number of 140 probably did not break down to turbulence like Roach reported, and was likely not reflective of what happened in the animal models. Now however, we now know that such models are extremely sensitive and have a somewhat limited applicability to the cardiovascular system (Bergersen et al., 2019; Haley et al., 2021).

Although the literature historically seems inconclusive from a mechanobiological and clinical perspective, a recent pre-clinical study by Koseki et al. found that aneurysm formation was caused by a combination of abnormally high WSS and "excessive mechanical stretch at a prospective site of IA formation" (Koseki et al., 2020). I.e., mechanical stretch came first, then followed by macrophage infiltration etc. Setting aside the abnormally high WSS that in isolation cannot explain aneurysm initiation, these results indeed indicate that the smooth muscle cells of the wall are 'highly sensitive to changes in applied loads' (Humphrey and Schwartz, 2021) and remodel adversely accordingly, potentially also at higher frequencies (Bittle, 1994). This is consistent with our previous reports and hypothesis of turbulent-like flows (and presumably wall vibrations) causing aneurysm formation in internal carotid arteries, which also contained energy at much higher frequencies (Valen-Sendstad et al., 2014). In conclusion, vascular wall vibrations may in fact be relevant as a mechanobiological stimuli within the wall, at least in pathological states.

4.3. Potential limitations

We simplistically here adopt the terminology of vibrations or mechanical stretch, previously reported to be associated with adverse wall remodelling (Roach,

1963a; Koseki et al., 2020). However, it is an open question whether there the actual mechanisms within the vascular wall are related to added circumferential stresses, shear stresses, or just radial vibrations. Neither do we claim that the vibration frequencies and amplitudes are 'patient-specific', only plausible. After all, the frequencies are on the low side compared to clinical observations (Kurokawa et al., 1994). There might be multiple reasons for that. First of all, the model we chose turned out to only be weakly unstable where the simulations "only" showed frequencies up to 160Hz in the flow, which is low also compared to the above-mentioned CFD studies. A mesh refinement study shown in Supplemental Material B didn't reveal any higher frequencies either. In addition to that, we also investigated the sensitivity of the solution to modeling parameters such as stiffness, wall thickness and flow rates, as shown in Supplemental Material C. The vibration amplitude was slightly affected by the stiffness, but the sensitivity study showed that the overall results were very robust. Although turtleFSI is a sophisticated numerical solver, and the most obvious limitation is the use of constant flow rate, which is physiologically unrealistic. I.e., both flow acceleration and deceleration in a pulsatile cardiac cycle are typically associated with flow stabilisation and destabilisation, respectively (Xu et al., 2020). However, the use of constant flow rate allows for easier isolation of flow instabilities and associated wall vibrations. What triggers "turbulence" here is just the sum of geometrical factors. Aneurysm wall heterogeneity, that can change material properties during disease progression, is another obvious issue (Cebral et al., 2017) that is not included in the mathematical model. Regarding the strain/stress curve, it can also be observed that our St. Venant-Kirchhoff constitutive law overestimates the stress state for a given stretch in comparison to the experimental data, which arguably provides a conservative estimate of the computed wall deformation. Although the global fit is acceptable, the stiffness is overestimated by a factor 2 in the strain regime below 1.15, but matches the stress/strain curves of intermediate aneurysm tissue reported by Costalat et al. (Costalat et al., 2011).

Another limitation is that we only had access to the lumen of the vascula-

ture, and the precise perivascular environment was unknown. That being said, the internal carotid artery in this model has a ”classical” shape and the arterial landmarks are easily identifiable (Bouthillier et al., 1996). The aneurysm in this model is located right after the opthalmic artery (absent in the model), which we know comes right after the carotid canal where the artery ”must” be clamped by bone. Assuming a fixed parent artery is therefore reasonable. Currently, ignoring the perianeurysmal environment is limitation for all aneurysm FSI simulations and it is possible that the cerebrospinal fluid could affect wall vibrations. Models of ”external tissue support” have been proposed and used for the aorta (Bäumler et al., 2020). Even there, where the momentum is much greater, it only affects the movement slightly when compared against medical images. Equivalently, we might need a model for ”external fluid support”. However, aneurysms do move or expand by approximately 1mm (Vanrossomme et al., 2015) throughout the cardiac cycle, and there is enough ”space” for aneurysms to move a distance equivalent of 4 times the wall thickness.

5. Conclusion

Our simulations suggest that turbulent-like flows can cause high-frequency aneurysm wall vibrations at hundreds of Hz, which is phenotypically consistent with both *in-vivo* clinical observations and *in-vitro* laboratory experiments. Vibrations could be relatively common in aneurysms and play a role in vascular remodeling, although the clear impact on mechanobiology remains to be investigated.

6. Acknowledgements

The study was supported by The Research Council of Norway through the SIMMIS project (262827). We acknowledge Dr. Adel M. Malek, MD, PhD at Tufts Medical Center, Boston, Massachusetts, for sharing the aneurysm model. The simulation were performed on the Experimental Infrastructure for Exploration of Exascale Computing (eX3), which is financially supported by the Re-

search Council of Norway under contract 270053. The authors would like to thank Aslak W. Bergersen, David A. Bruneau and David A. Steinman for fruitful discussions.

References

Alperin, N.J., Lee, S.H., Loth, F., Raksin, P.B., Lichtor, T., 2000. Mr-intracranial pressure (icp): a method to measure intracranial elastance and pressure noninvasively by means of mr imaging: baboon and human study. Radiology 217, 877–885.

Antiga, L., Piccinelli, M., Botti, L., Ene-Iordache, B., Remuzzi, A., Steinman, D.A., 2008. An image-based modeling framework for patient-specific computational hemodynamics. Medical & biological engineering & computing 46, 1097–1112.

Arzani, A., Shadden, S.C., 2018. Wall shear stress fixed points in cardiovascular fluid mechanics. Journal of biomechanics 73, 145–152.

Baek, H., Jayaraman, M.V., Richardson, P.D., Karniadakis, G.E., 2010. Flow instability and wall shear stress variation in intracranial aneurysms. Journal of The Royal Society Interface 7, 967–88.

Balasso, A., Fritzsche, M., Liepsch, D., Prothmann, S., Kirschke, J.S., Sindeev, S., Frolov, S., Friedrich, B., 2018. High-frequency wall vibrations in a cerebral patient-specific aneurysm model. Biomedical Engineering/Biomedizinische Technik .

Bäumler, K., Vedula, V., Sailer, A.M., Seo, J., Chiu, P., Mistelbauer, G., Chan, F.P., Fischbein, M.P., Marsden, A.L., Fleischmann, D., 2020. Fluid–structure interaction simulations of patient-specific aortic dissection. Biomechanics and modeling in mechanobiology 19, 1607–1628.

Berg, P., Voß, S., Saalfeld, S., Janiga, G., Bergersen, A.W., Valen-Sendstad, K., Bruening, J., Goubergrits, L., Spuler, A., Cancelliere, N.M., et al., 2018.

Multiple aneurysms anatomy challenge 2018 (match): phase i: segmentation. Cardiovascular engineering and technology 9, 565–581.

Bergersen, A.W., Mortensen, M., Valen-Sendstad, K., 2019. The fda nozzle benchmark: 'in theory there is no difference between theory and practice, but in practice there is'. International journal for numerical methods in biomedical engineering 35, e3150.

Bergersen, A.W., Slyngstad, A., Gjertsen, S., Souche, A., Valen-Sendstad, K., 2020. turtlefsi: A robust and monolithic fenics-based fluid-structure interaction solver. Journal of Open Source Software 5, 2089.

Bittle, B.B., 1994. An investigation into the role of arterial wall vibration in the pathogenesis of atherosclerosis. Iowa State University.

Bols, J., Degroote, J., Trachet, B., Verhegghe, B., Segers, P., Vierendeels, J., 2013. A computational method to assess the in vivo stresses and unloaded configuration of patient-specific blood vessels. Journal of computational and Applied mathematics 246, 10–17.

Bouthillier, A., Van Loveren, H.R., Keller, J.T., 1996. Segments of the internal carotid artery: a new classification. Neurosurgery 38, 425–433.

Cebral, J., Ollikainen, E., Chung, B.J., Mut, F., Sippola, V., Jahromi, B.R., Tulamo, R., Hernesniemi, J., Niemelä, M., Robertson, A., et al., 2017. Flow conditions in the intracranial aneurysm lumen are associated with inflammation and degenerative changes of the aneurysm wall. American Journal of Neuroradiology 38, 119–126.

Cebral, J.R., Mut, F., Weir, J., Putman, C., 2011. Quantitative characterization of the hemodynamic environment in ruptured and unruptured brain aneurysms. American Journal of Neuroradiology 32, 145–151.

Chien, S., 2007. Mechanotransduction and endothelial cell homeostasis: the wisdom of the cell. Am J Physiol Heart Circ Physiol

292, H1209–1224. URL: http://ajpheart.physiology.org/cgi/content/abstract/292/3/H1209, doi:10.1152/ajpheart.01047.2006, arXiv:http://ajpheart.physiology.org/cgi/reprint/292/3/H1209.pdf.

Costalat, V., Sanchez, M., Ambard, D., Thines, L., Lonjon, N., Nicoud, F., Brunel, H., Lejeune, J.P., Dufour, H., Bouillot, P., Lhaldky, J.P., Kouri, K., Segnarbieux, F., Maurage, C.a., Lobotesis, K., Villa-Uriol, M.C., Zhang, C., Frangi, a.F., Mercier, G., Bonafé, a., Sarry, L., Jourdan, F., 2011. Biomechanical wall properties of human intracranial aneurysms resected following surgical clipping (IRRAs Project). Journal of biomechanics 44, 2685–91. URL: http://www.ncbi.nlm.nih.gov/pubmed/21924427, doi:10.1016/j.jbiomech.2011.07.026.

Ferguson, G.G., 1970. Turbulence in human intracranial saccular aneurysms. Journal of Neurosurgery 33, 485–97.

Fernández, M.A., 2011. Coupling schemes for incompressible fluid-structure interaction: implicit, semi-implicit and explicit. SeMA Journal 55, 59–108.

Ford, M.D., Piomelli, U., 2012. Exploring high frequency temporal fluctuations in the terminal aneurysm of the basilar bifurcation. Journal of biomechanical engineering 134, 091003.

Frösen, J., Piippo, A., Paetau, A., Kangasniemi, M., Niemelä, M., Hernesniemi, J., Jääskeläinen, J., 2004. Remodeling of saccular cerebral artery aneurysm wall is associated with rupture: histological analysis of 24 unruptured and 42 ruptured cases. Stroke; a journal of cerebral circulation 35, 2287–93.

Haley, A., Valen-Sendstad, K., Steinman, D., 2021. On delayed transition to turbulence in an eccentric stenosis model for clean vs. noisy high-fidelity cfd. Journal of Biomechanics 125, 110588.

Hoi, Y., Wasserman, B.A., Xie, Y.J., Najjar, S.S., Ferruci, L., Lakatta, E.G., Gerstenblith, G., Steinman, D.A., 2010. Characterization of volumetric flow

rate waveforms at the carotid bifurcations of older adults. Physiological Measurement 31, 291.

Humphrey, J.D., Schwartz, M.A., 2021. Vascular mechanobiology: homeostasis, adaptation, and disease. Annual Review of Biomedical Engineering 23, 1–27.

Hung, E.J.n., Botwin, M.R., 1975. Mechanics of rupture of cerebral saccular aneurysms. Journal of biomechanics 8, 385–392.

Hunter, J.D., 2007. Matplotlib: A 2d graphics environment. Computing in Science & Engineering 9, 90–95. doi:10.1109/MCSE.2007.55.

Isaksen, J.r.G., Bazilevs, Y., Kvamsdal, T., Zhang, Y., Kaspersen, J.H., Waterloo, K., Romner, B., Ingebrigtsen, T., 2008. Determination of wall tension in cerebral artery aneurysms by numerical simulation. Stroke; a journal of cerebral circulation 39, 3172–8. URL: http://www.ncbi.nlm.nih.gov/pubmed/18818402, doi:10.1161/STROKEAHA.107.503698.

Jou, L.D., Mawad, M.E., 2010. Indirect measurement of aneurysm wall thickness using digital stethoscope. Neurological research 32, 661–5. URL: http://www.ncbi.nlm.nih.gov/pubmed/19660238, doi:10.1179/016164109X12464612122777.

Khan, M., Toro Arana, V., Najafi, M., MacDonald, D., Natarajan, T., Valen-Sendstad, K., Steinman, D., 2021. On the prevalence of flow instabilities from high-fidelity computational fluid dynamics of intracranial bifurcation aneurysms. Journal of Biomechanics , 110683URL: https://www.sciencedirect.com/science/article/pii/S0021929021004528, doi:https://doi.org/10.1016/j.jbiomech.2021.110683.

Koseki, H., Miyata, H., Shimo, S., Ohno, N., Mifune, K., Shimano, K., Yamamoto, K., Nozaki, K., Kasuya, H., Narumiya, S., et al., 2020. Two diverse hemodynamic forces, a mechanical stretch and a high wall shear stress, determine intracranial aneurysm formation. Translational stroke research 11, 80–92.

Kurokawa, Y., Abiko, S., Watanabe, K., 1994. Noninvasive detection of intracranial vascular lesions by recording blood flow sounds. Stroke 25, 397–402. arXiv:http://stroke.ahajournals.org/cgi/reprint/25/2/397.pdf.

Liang, L., Steinman, D.A., Brina, O., Chnafa, C., Cancelliere, N.M., Pereira, V.M., 2019. Towards the clinical utility of cfd for assessment of intracranial aneurysm rupture–a systematic review and novel parameter-ranking tool. Journal of neurointerventional surgery 11, 153–158.

Malek, A.M., Alper, S., Izumo, S., 1999. Hemodynamic Shear Stress and Its Role in Atherosclerosis. JAMA 282, 2035–2042.

Ojha, M., Langille, B.L., 1993. Evidence that turbulence is not the cause of poststenotic dilatation in rabbit carotid arteries. Arteriosclerosis and thrombosis: a journal of vascular biology 13, 977–984.

PB, D., 1991. Poststenotic dilatation. Surg Gynecol Obstet 172(6), 503–8.

Pope, S.B., 2000. Turbulent Flows. Cambridge University Press.

Richardson, C., Kofman, O., 1951. Cranial bruit intracranial saccular aneurysms. Transactions of the American Neurological Association 56, 151–154.

Rinkel, G.J.E., Djibuti, M., Algra, A., van Gijn, J., 1998. Prevalence and Risk of Rupture of Intracranial Aneurysms : A Systematic Review. Stroke 29, 251–256. arXiv:http://stroke.ahajournals.org/cgi/reprint/29/1/251.pdf.

Roach, M.R., 1963a. Changes in arterial distensibility as a cause of poststenotic dilatation. The American journal of cardiology 12, 802–815.

Roach, M.R., 1963b. An experimental study of the production and time course of poststenotic dilatation in the femoral and carotid arteries of adult dogs. Circulation Research 13, 537–551.

Robertson, A., Watton, P., 2012. Computational fluid dynamics in aneurysm research: critical reflections, future directions. American journal of neuroradiology 33, 992–995.

Robertson, A.M., Duan, X., Aziz, K.M., Hill, M.R., Watkins, S.C., Cebral, J.R., 2015. Diversity in the strength and structure of unruptured cerebral aneurysms. Annals of biomedical engineering 43, 1502–1515.

Shojima, M., Oshima, M., Takagi, K., Torii, R., Hayakawa, M., Katada, K., Morita, A., Kirino, T., 2004. Magnitude and role of wall shear stress on cerebral aneurysm computational fluid dynamic study of 20 middle cerebral artery aneurysms. Stroke 35, 2500–2505.

Steinman, D., Hoi, Y., Fahy, P., Morris, L., N, M.W., Aristokleous, Anayiotos, A., Papaharilaou, Y., Arzani, A., Shadden, S., Berg, P., Janiga, G., Bols, J., Segers, P., Bressloff, N., Cibis, M., Gijsen, F., Cito, S., Pallars, J., Browne, L., Costelloe, J., Lynch, A., Degroote, J., Vierendeels, J., Fu, W., QiaoA, A., Hodis, S., Kallmes, D., Kalsi, H., Long, Q., Kheyfets, V., Finol, E., Kono, K., Malek, A., Lauric, A., Menon, P., Pekkan, K., Moghadam, M.E., Marsden, A., Oshima, M., V, K.K., Peiffer, Mohamied, Y., Sherwin, S., Schaller, J., Goubergrits, L., Usera, G., Mendina, M., Valen-Sendstad, K., Habets, D., Xiang, J., Meng, H., Yu, Y., Karniadakis, G., Shaffer, N., Loth, F., 2013. Variability of Computational Fluid Dynamics Solutions for Pressure and Flow in a Giant Aneurysm The ASME 2012 Summer Bioengineering Conference CFD Challenge. Journal of Biomedical Engineering 135.

Steinman, D.A., Milner, J.S., Norley, C.J., Lownie, S.P., Holdsworth, D.W., 2003. Image-based computational simulation of flow dynamics in a giant intracranial aneurysm. American Journal of Neuroradiology 24, 559–566.

Steinman, D.A., Pereira, V.M., 2019. How patient specific are patient-specific computational models of cerebral aneurysms? an overview of sources of error and variability. Neurosurgical focus 47, E14.

Strother, C.M., Graves, V.B., Rappe, A., 1992. Aneurysm hemodynamics: an experimental study. American Journal of Neuroradiology 13, 1089–1095.

Tennekes, H., Lumley, J.L., 1972. A First Course in Turbulence. MIT Press. URL: http://mitpress.mit.edu/catalog/item/default.asp?ttype=2&tid=4658.

Torii, R., Oshima, M., Kobayashi, T., Takagi, K., Tezduyar, T.E., 2007. Influence of wall elasticity in patient-specific hemodynamic simulations. Computers & Fluids 36, 160–168. URL: http://linkinghub.elsevier.com/retrieve/pii/S0045793005001374, doi:10.1016/j.compfluid.2005.07.014.

Ujiie, H., et al., 1999. Effects of size and shape (aspect ratio) on the hemodynamics of saccular aneurysms: a possible index for surgical treatment of intracranial aneurysms. Neurosurgery 45, 119–130.

Valen-Sendstad, K., Bergersen, A.W., Shimogonya, Y., Goubergrits, L., Bruening, J., Pallares, J., Cito, S., Piskin, S., Pekkan, K., Geers, A.J., et al., 2018. Real-world variability in the prediction of intracranial aneurysm wall shear stress: the 2015 international aneurysm cfd challenge. Cardiovascular engineering and technology 9, 544–564.

Valen-Sendstad, K., Mardal, K.A., Logg, A., 2012. Computational hemodynamics. Springer. volume 84 of *Lecture Notes in Computational Science and Engineering,*. chapter 23. pp. 439–454.

Valen-Sendstad, K., Mardal, K.A., Mortensen, M., Reif, B.A.P., Langtangen, H.P., 2011. Direct Numerical Simulation of Transitional Flow in a Patient–Specific Intracranial Aneurysm. Journal of Biomechanics 44, 2826–2832.

Valen-Sendstad, K., Piccinelli, M., KrishnankuttyRema, R., Steinman, D.A., 2015. Estimation of inlet flow rates for image-based aneurysm cfd models: Where and how to begin? Annals of biomedical engineering 43, 1422–1431.

Valen-Sendstad, K., Piccinelli, M., Steinman, D.A., 2014. High-resolution computational fluid dynamics detects flow instabilities in the carotid siphon: Implications for aneurysm initiation and rupture? Journal of biomechanics 47, 3210–3216.

Valen-Sendstad, K., Steinman, D.A., 2014. Mind the gap: Impact of CFD solution strategy on prediction of intra-cranial aneurysm hemodynamics and rupture status. AJNR. American Journal of Neuroradiology 35, 536–543. Commentary, 544-545.

Vanrossomme, A., Eker, O.F., Thiran, J.P., Courbebaisse, G., Boudjeltia, K.Z., 2015. Intracranial aneurysms: Wall motion analysis for prediction of rupture. American journal of neuroradiology 36, 1796–1802.

Wick, T., 2011. Adaptive finite element simulation of fluid-structure interaction with application to heart-valve dynamics. Ph.D. thesis.

Wiebers et al., 2003. Unruptured intracranial aneurysms: natural history, clinical outcome, and risks of surgical and endovascular treatment. The Lancet 362, 103–110.

Xiang, J., Natarajan, S.K., Tremmel, M., Ma, D., Mocco, J., Hopkins, L.N., Siddiqui, A.H., Levy, E.I., Meng, H., 2011. Hemodynamic-Morphologic Discriminants for Intracranial Aneurysm Rupture. Stroke 42, 144–152.

Xu, D., Varshney, A., Ma, X., Song, B., Riedl, M., Avila, M., Hof, B., 2020. Nonlinear hydrodynamic instability and turbulence in pulsatile flow. Proceedings of the National Academy of Sciences 117, 11233–11239.

Yagi, T., Ishida, F., Shojima, M., Anzai, H., Fujimura, S., Sano, T., Shinozaki, S., Yamanaka, Y., Yamamoto, Y., Okamoto, Y., et al., 2019. Systematic review of hemodynamic discriminators for ruptured intracranial aneurysms. Journal of Biorheology 33, 53–64.

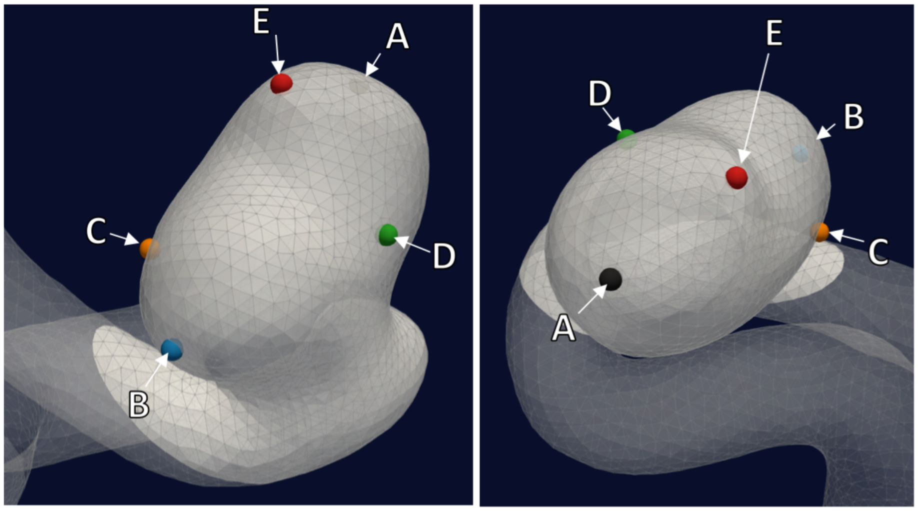

Figure 1: Two views of the aneurysm model and mesh with rigid (opaque) and compliant (translucent) wall domains. The coloured dots correspond to the five probe approximate locations of the fluid, pressure, and deformation, shown later in the Results.

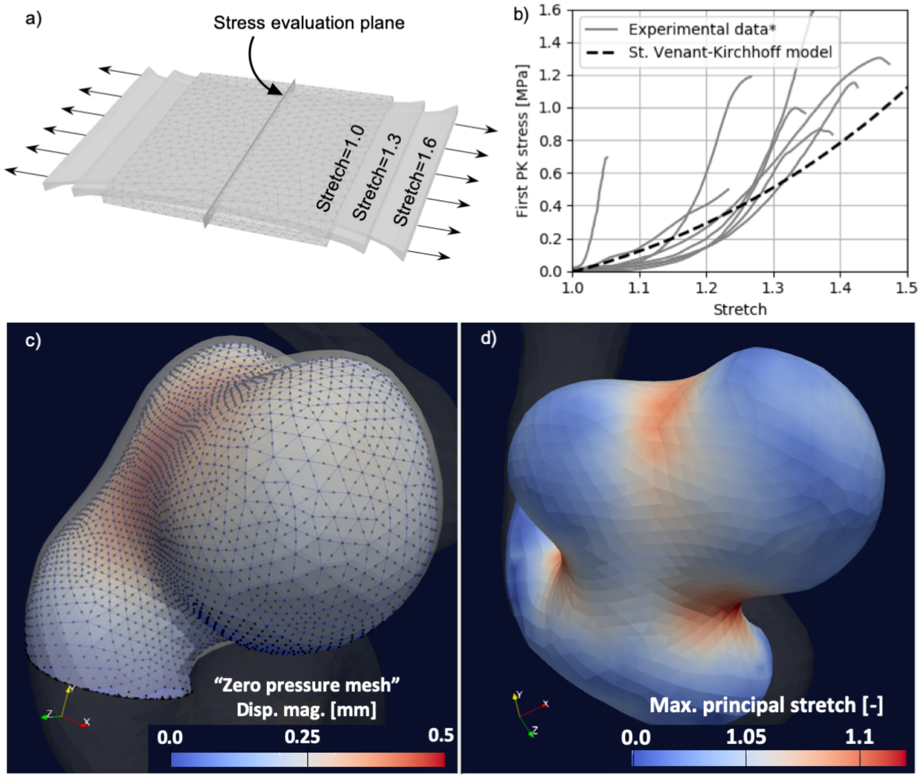

Figure 2: a) Numerically computed uniaxial test at 3 different stretch levels. b) Comparison between experimental and numerical constitutive laws obtained from uniaxial test. c) The geometry (shaded) versus the zero pressure geometry (inner mesh) color coded by the equivalent deformation field. d) Estimated maximal principal stretch representative of the prestressed model.

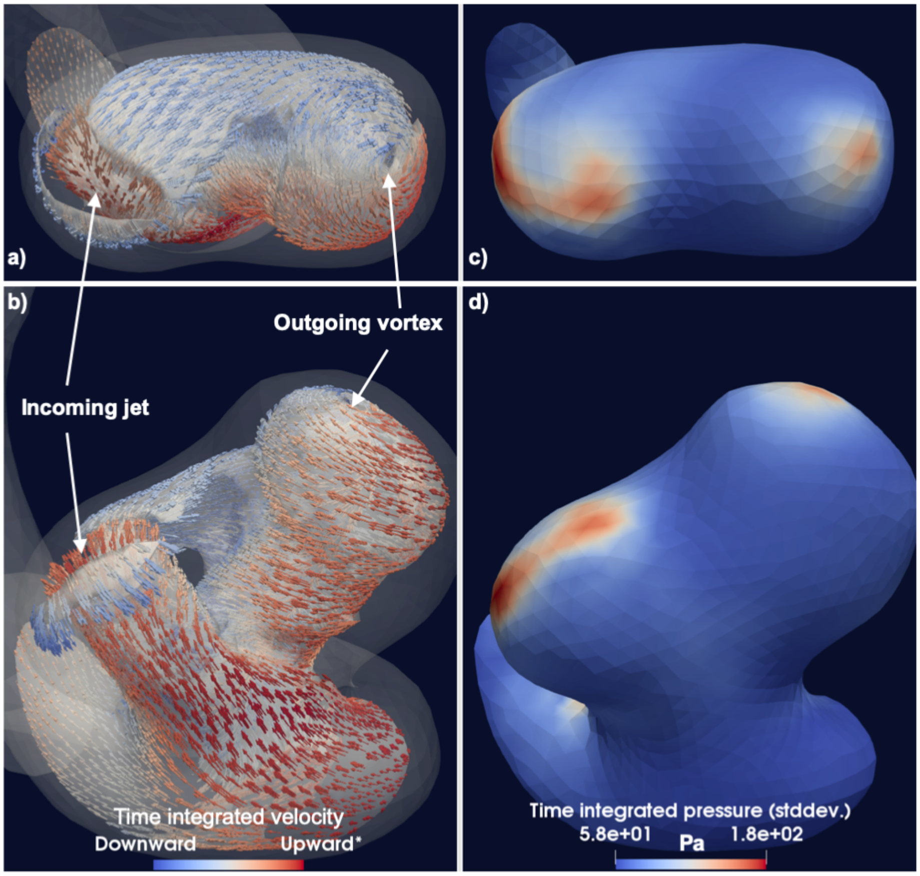

Figure 3: a-b) Time integrated flow field visualized with velocity arrows along the 0.58 mm/ms iso-surface velocity magnitude. The red/blue color-map of the velocity arrows corresponds, respectively, to the upward/downward pointing direction of the aneurysm top view perspective a). c-d) Standard deviation of the time integrated pressure field plotted along the inner surface of the aneurysm wall.

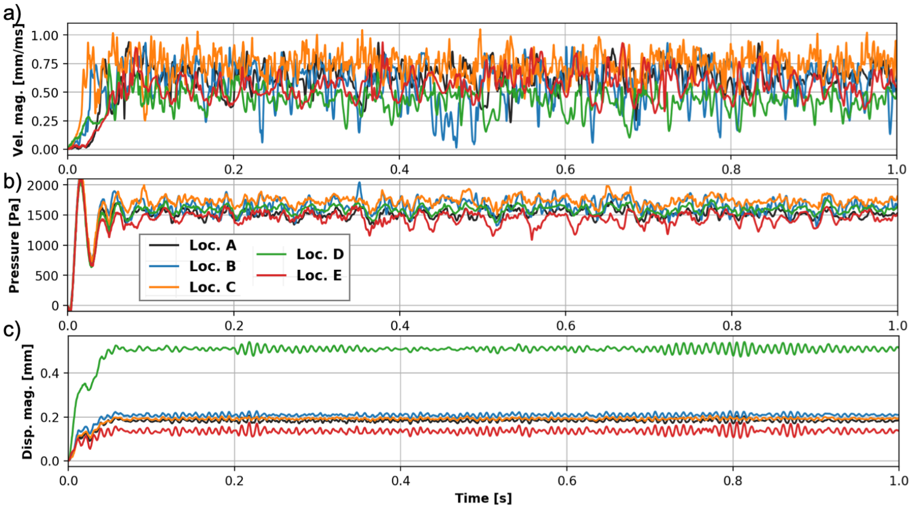

Figure 4: Temporal evolution of the fluid velocity in a, the fluid pressure in b, and the wall deformation c at the locations indicated in Figure 1.

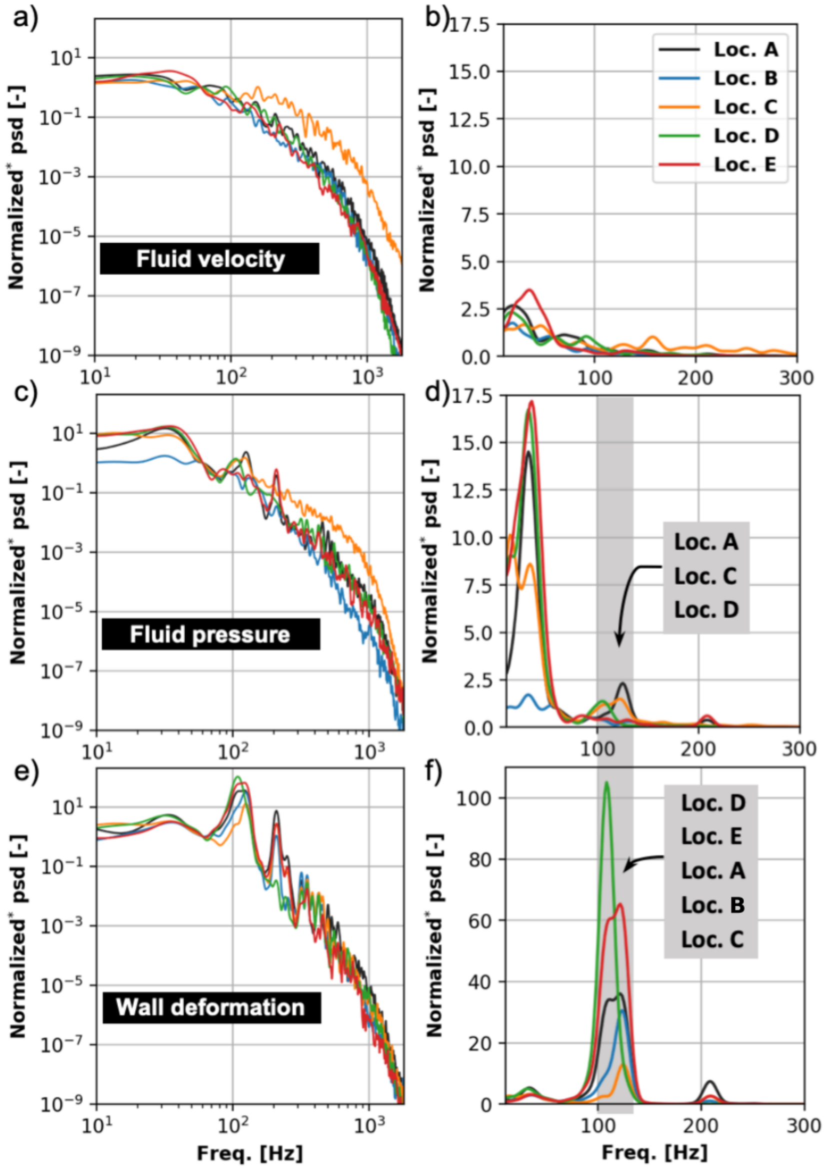

Figure 5: Power spectrum decomposition (PSD) of the temporal signals of fluid velocity, pressure, and wall deformation, respectively, extracted from locations specified in Figure 1. Data plotted on logarithmic scale axis (left) and linear scale axis (right).
